# Variation in *Leishmania* chemokine suppression driven by diversification of the GP63 virulence factor

**DOI:** 10.1371/journal.pntd.0009224

**Published:** 2021-10-28

**Authors:** Alejandro L. Antonia, Alyson B. Barnes, Amelia T. Martin, Liuyang Wang, Dennis C. Ko

**Affiliations:** 1 Department of Molecular Genetics and Microbiology, School of Medicine, Duke University, Durham, North Carolina, United States of America; 2 Division of Infectious Diseases, Department of Medicine, School of Medicine, Duke University, Durham, North Carolina, United States of America; Insitut Pasteur de Tunis, TUNISIA

## Abstract

Leishmaniasis is a neglected tropical disease with diverse outcomes ranging from self-healing lesions, to progressive non-healing lesions, to metastatic spread and destruction of mucous membranes. Although resolution of cutaneous leishmaniasis is a classic example of type-1 immunity leading to self-healing lesions, an excess of type-1 related inflammation can contribute to immunopathology and metastatic spread. *Leishmania* genetic diversity can contribute to variation in polarization and robustness of the immune response through differences in both pathogen sensing by the host and immune evasion by the parasite. In this study, we observed a difference in parasite chemokine suppression between the *Leishmania (L*.*)* subgenus and the *Viannia (V*.*)* subgenus, which is associated with severe immune-mediated pathology such as mucocutaneous leishmaniasis. While *Leishmania (L*.*)* subgenus parasites utilize the virulence factor and metalloprotease glycoprotein-63 *(gp63)* to suppress the type-1 associated host chemokine CXCL10, *L*. *(V*.*) panamensis* did not suppress CXCL10. To understand the molecular basis for the inter-species variation in chemokine suppression, we used *in silico* modeling to identify a putative CXCL10-binding site on GP63. The putative CXCL10 binding site is in a region of *gp63* under significant positive selection, and it varies from the *L*. *major* wild-type sequence in all *gp63* alleles identified in the *L*. *(V*.*) panamensis* reference genome. Mutating wild-type *L*. *(L*.*) major gp63* to the *L*. *(V*.*) panamensis* sequence at the putative binding site impaired cleavage of CXCL10 but not a non-specific protease substrate. Notably, *Viannia* clinical isolates confirmed that *L*. *(V*.*) panamensis* primarily encodes non-CXCL10-cleaving *gp63* alleles. In contrast, *L*. *(V*.*) braziliensis* has an intermediate level of activity, consistent with this species having more equal proportions of both alleles. Our results demonstrate how parasite genetic diversity can contribute to variation in immune responses to *Leishmania* spp. infection that may play critical roles in the outcome of infection.

## Author summary

Leishmaniasis is a neglected tropical disease caused by *Leishmania* parasites and spread by the bites of infected sand flies. Most cases of leishmaniasis present as self-healing sores that are resolved by a balanced immune response. Other cases of leishmaniasis involve spread to sites distant from the original bite, including damage of the inner surfaces of the mouth and nose. These cases of leishmaniasis involve an excessive immune response. *Leishmania* parasites produce virulence factor proteins, such as GP63, to trick the immune system into mounting a weaker response. GP63 specifically degrades signaling proteins that attract and activate certain immune cells. Here, we demonstrate that *Leishmania* parasite species have evolved to differ in their ability to degrade signaling proteins. In *Leishmania* species known to cause more immune-mediated tissue damage, the GP63 virulence factor has evolved to not degrade specific immune signaling proteins, thus attracting, and activating more immune cells. Our results demonstrate how diversity among *Leishmania* parasite species can contribute to variation in immune responses that may play critical roles in the outcome of infection.

## Introduction

Parasites in the genus *Leishmania* infect over 1.6 million people annually causing a diverse collection of diseases ranging from visceral systemic illness to simple self-resolving cutaneous lesions to diffuse non-healing lesions with metastatic spread [1,2]. This diverse spectrum of disease outcomes is in part mediated by parasite genetic diversity influencing the host-immune response. Due to the high psychological and social impact caused by disfiguring skin lesions [3], and current drug options limited by high prices and severe side effects [4], improved understanding of the mechanisms underlying differential disease outcome is paramount.

Although cutaneous leishmaniasis canonically requires T-helper 1 polarization for lesion resolution, improved understanding of *Leishmania* parasite diversity has led to the elucidation of multiple exceptions to this rule. Experiments using the murine model of leishmaniasis defined the T-helper (T_h_) cell polarization dichotomy where T_h_1 polarization in C57BL6 mice protects against cutaneous leishmaniasis and T_h_2 polarization in BALB/c mice leads to progressive non-healing infections [5,6]. Similarly, humans with self-healing lesions have higher levels of T_h_1 associated cytokines [7–9]. However, studies in mice and humans have revealed instances where type 1 immune responses are not protective against cutaneous leishmaniasis. For example, mice infected with the *L*. *(L*.*) major* Seidman strain develop non-healing lesions despite robust T_h_1 polarization [10,11]. Additionally, patients infected with parasites belonging to the *Viannia* subgenus of parasites have lesions characterized by significantly elevated expression of type-1 associated genes such as *IFN*-*γ*, *Granzyme-B* and *CXCL10* [12–14]. Further, these markers of type-1 associated immunopathology are exacerbated by infection of the parasite with the *Leishmania* RNA virus (LRV1) [15] or co-infection of the mouse with lymphocytic choriomeningitis virus (LCMV) [16,17]. These exceptions to the rule of protective type-1 immunity raise the question: what determines if a type-1 immune response protects the patient or exacerbates disease?

Clues to answering this question may come from understanding *Leishmania* strategies to evade the host immune response [18]. *Leishmania* spp. parasites evade the host immune response by diverse mechanisms including inhibition of phagolysosome development, antigen cross-presentation, and intracellular macrophage signaling (reviewed in Gupta et al. 2013 [19]). One virulence factor involved in multiple mechanisms of immune evasion is the matrix-metalloprotease glycoprotein-63 (*gp63*) [20], which is able to cleave host substrates involved in anti-parasitic defense including complement C3b [21], the myristoylated alanine rich C kinase substrate (MARCKS) [22], and CXCL10 [23]. These mechanisms interfere with the host response after infection thereby modulating the balance between protection and pathogenesis.

Parasite genetic diversity also drives differences in disease outcome through both host pathogen sensing and pathogen immune evasion. Although there is a high degree of conservation and synteny between parasite strains, differences in genetic structural elements such as gene duplications and losses, transposable elements, and pseudogenes are thought to play a role in mediating distinct disease outcomes [24–26]. These differences are highlighted within the *Viannia* subgenus, which is associated with lesions characterized by host-cytotoxic immunopathology. Differences in the structure of the lipophosphoglycans produced by *L*. *(V*.*) braziliensis* and *L*. *(L*.*) infantum* lead to different patterns of toll-like receptor (TLR) activation [27]. Beyond sensing, parasite genetic variation facilitates the generation of diverse immune evasion strategies. For example, parasites in the *Viannia* subgenus have undergone a significant expansion in copy number corresponding with an increase in genetic variation of *gp63* [28–31]. This diversification correlates with variation in *gp63* expression and the downstream effect of phagolysosome maturation between different *L*. *(V*.*) braziliensis* isolates [32]. Furthermore, evolutionary studies have identified a region of positive selection around the active site hypothesized to alter substrate specificity [33,34]. However, specific genetic polymorphisms responsible for driving observed phenotypic differences between parasites that cause distinct forms of disease have yet to be established.

In this study, we characterize how genetic diversity results in variation in function of the glycoprotein-63 virulence factor between the *Leishmania* and *Viannia* subgenera. We observed that *Viannia* parasites have reduced capacity to cleave CXCL10 by GP63, highlighted by complete loss of cleavage by *L*. (*V*.) *panamensis*. To define how interspecies *gp63* variation results in loss of CXCL10 cleavage, we used a combination of modeling protein-protein interactions and identification of sites under evolutionary pressure. First, protein-protein modeling of the GP63-CXCL10 interaction revealed a putative CXCL10 binding site that is mutated from D463 to N463 preferentially in the *Viannia* subgenus. Screening additional *Viannia* species demonstrated that parasites containing both alleles have an intermediate CXCL10 suppression phenotype. Subsequently, using the mixed effects model of evolution (MEME) we found that 4 out of 79 individual amino acids under episodic positive selection are located within 5 amino acid residues of the predicted CXCL10 binding site. Finally, site-directed mutagenesis of *L*. (*L*.) *major gp63* at the predicted CXCL10 binding site (D463N), as well as adjacent residues under significant episodic positive selection, confirmed this region is involved in cleavage of CXCL10 but not the nonspecific substrate azocasein. Understanding how genetic diversity of GP63 and other virulence factors lead to differences in immune phenotypes between divergent *Leishmania* spp. will be critical in the design and proper application of novel therapies for the prevention and treatment of leishmaniasis.

## Results

### *Glycoprotein-63 produced by* L. (V.) panamensis (PSC-1) does not cleave human CXCL10

**Fig 1:**
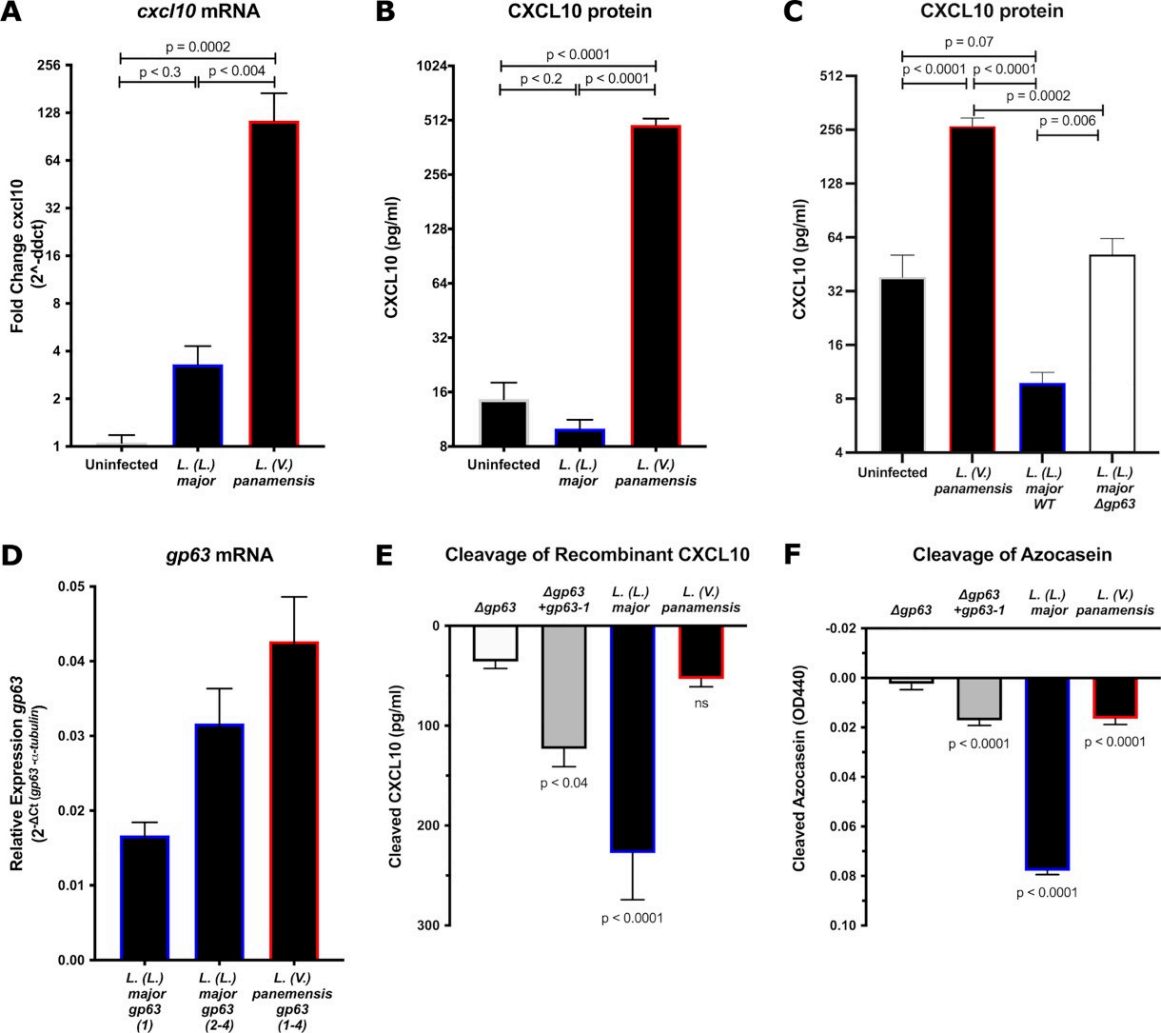
*Leishmania Viannia panamensis* lacks glycoprotein-63 dependent cleavage of human CXCL10. *(A)* L. (L.) major *and* L. (V.) panamensis *infection results in increased CXCL10 transcript in THP-1 monocytes*. PMA-differentiated THP-1 monocytes were infected with *L*. *(L*.*) major* Friedlin or *L*. *(V*.*) panamensis* PSC-1 at a multiplicity of infection (MOI) of 6 for 24 hours. mRNA was quantified by RT-PCR for human *CXCL10* relative to *rRNA45s5* housekeeping gene by ΔΔC_t_. Average fold change (2^-ΔΔCt^) +/- standard error of the mean (SEM) is plotted for 8–9 biological replicates across three independent experiments. One outlier was removed from the *L*. *(L*.*) major* condition as identified by the ROUT method with Q set to 0.1%. P-values calculated by one-way ANOVA with Holm-Sidak post-hoc test on C_t_ values. *(B)* L. (L.) major *but not* L. (V.) panamensis *infection suppresses CXCL10 protein in THP1 monocytes*. CXCL10 protein was measured from the supernatants of infected PMA-differentiated THP-1 monocytes 24 hours post infection by ELISA. Samples below the ELISA range were set to the lower limit of detection (7.8125 pg/mL). Mean +/- SEM is plotted for 8–9 biological replicates across three independent experiments. One outlier was removed from the *L*. *(L*.*) major* condition as identified by the ROUT method with Q set to 0.1%. P-values calculated by one-way ANOVA with Holm-Sidak post hoc test on log_2_([CXCL10]). *(C)* L. (L.) major *suppression of CXCL10 is mediated by gp63 in THP-1 monocytes*. PMA-differentiated THP-1 monocytes were infected with *L*. *(V*.*) panamensis* PSC-1, *L*. *(L*.*) major* Seidman wild-type (WT), or *L*. *(L*.*) major* Seidman *Δgp63* at an MOI of 6 for 48 hours. CXCL10 protein was measured from the supernatants by ELISA. Samples below the ELISA range were set to the lower limit of detection (7.8125 pg/mL for experiment 1, 3.91 pg/ml for experiment 2). Mean +/- SEM is plotted for 8 biological replicates across two independent experiments. P-values calculated by one-way ANOVA with Holm-Sidak post hoc test on log_2_([CXCL10]). *(D)* L. (L.) major *and* L. (V.) panamensis *both express* gp63 *mRNA at the time of infection*. Parasite mRNA was obtained from parasites incubated at 37°C for 1 hour. mRNA was quantified by RT-PCR for *Leishmania gp63* relative to *α-tubulin* housekeeping gene by ΔΔC_t._ For primer design to quantify *gp63* expression in different species: *L*. *(L*.*) major* Friedlin *gp63* required two sets of primers to amplify four copies of *gp63* due to sequence variation (primer 1 amplifies *LmjF_10*.*0470*, and the primers for 2–4 amplify *LmjF_10*.*0460*, *LmjF_10*.*0465*, *LmjF_10*.*0480*), whereas *L*. *(V*.*) panamensis* required a single set of primers due to greater homology between four unique copies (primers for copy 1–4 amplify *LPMP_100410*, *LPMP_100420*, *LPMP_100430*, *LPMP_100440*). Relative expression (2^-ΔΔCt^) is plotted as mean +/- SEM for three biological replicates across three independent experiments. *(E)* L. (L.) major *but not* L. (V.) panamensis *cleaves human recombinant CXCL10*. 1x10^6^ parasites were incubated with 500 pg/mL of human recombinant CXCL10 at 37°C for 1 hour prior to measuring the remaining CXCL10 by ELISA. Cleaved CXCL10 was calculated by subtracting the CXCL10 in the parasite conditions from a no-parasite media control. Mean cleaved CXCL10 +/- SEM is plotted from 10 biological replicates across 5 independent experiments. *(F)* L. (L.) major *and* L. (V.) panamensis *both cleave the non-specific colorimetric protease substrate azocasein*. 5x10^7^ parasites were incubated with 50 mg/mL of azocasein at 37°C for 5 hours. Cleaved azocasein was determined by subtracting OD440 from the no-parasite media control. Mean OD440 of cleaved azocasein +/- SEM is plotted from 7 biological replicates across 5 independent experiments. For (D) and (E), P-values calculated by one-way ANOVA comparing all samples to the Δ*gp63* control by Holm-Sidak post-hoc test.

As *Viannia* parasites induce high c*xcl10* expression [12,13,15] and have a large copy number expansion [28,30] of the CXCL10-cleaving protease GP63 [23], we asked whether *Leishmania Viannia panamensis* infection results in a net increase or decrease of CXCL10 protein. PMA-differentiated THP-1 macrophages infected with *L*. (*L*.) *major* began to increase host *cxcl10* transcription early at 24 hours post infection (p<0.3), consistent with multiple previous studies demonstrating increased *CXCL10* mRNA after infection [23,35]. However, *L*. *(L*.*) major* (Freidlin strain) infection completely suppressed the induction at the protein level (Fig 1A and 1B), consistent with our previous observations [23]. In contrast, despite the large copy number expansion of *gp63* in *Viannia* parasites, *L*. (*V*.) *panamensis* infection resulted in higher induction of *cxcl10* transcript and no suppression of CXCL10 protein (Fig 1A and 1B). Indeed, in THP-1 macrophages, *L*.*(V*.*) panamensis* infection resulted in greater CXCL10 compared to the previously generated *L*. *(L*.*) major* (Seidman strain) gp63 knockout (Δ*gp63*; [21]): *L*. *(L*.*) major* (Seidman strain) wild-type infection resulted in CXCL10 near the lower limit of detection, *L*. *(L*.*) major* (Seidman strain) *Δgp63* had significantly increased CXCL10 protein, and *L*. *(V*.*) panamensis* resulted in even greater levels of CXCL10 protein (Fig 1C).

An inability of *L*. *(V*.*) panamensis* to overcome the increased *cxcl10* transcript could be due to either a lack of *gp63* expression or a reduction in GP63 cleavage activity. To compare *gp63* expression, we measured *gp63* mRNA by *L*. (*V*.) panamensis and *L*. *(L*.*) major* in cultured promastigotes at the time of infection. The two *Leishmania* spp. expressed comparable *gp63* mRNA (Fig 1D), suggesting that differences in gene expression do not account for the observed lack of CXCL10 suppression by *L*. *(V*.*) panamensis*. To compare the ability of *L*. (*V*.) *panamensis* and *L*. *(L*.*) major* to cleave equivalent amounts of recombinant CXCL10 and control for variation in host chemokine production, we incubated parasites with human recombinant CXCL10. *L*. *(L*.*) major* that is *gp63* deficient (Δ*gp63*) and complemented (Δ*gp63+gp63-1*) were included as controls. Wildtype *L*. (*L*.) *major* cleaved the majority of CXCL10 by 1 hour; however, there was no significant reduction of CXCL10 by *L*. (*V*.) *panamensis* relative to the Δ*gp63* strain (Fig 1E). Next, we tested whether this was due to a complete loss of proteolytic activity or a change in substrate-specific activity by repeating the cleavage assay with the non-specific colorimetric substrate azocasein. *L*. (*V*.) *panamensis* cleaved significantly more azocasein than the Δ*gp63* strain, although still had reduced activity relative to wild-type *L*. (*L*.) *major* (Fig 1F). Therefore, the lack of CXCL10 suppression by *L*. *(V*.*) panamensis* is due to loss of substrate-specific enzymatic activity of GP63.

### Identification of predicted CXCL10 binding site on GP63

**Fig 2:**
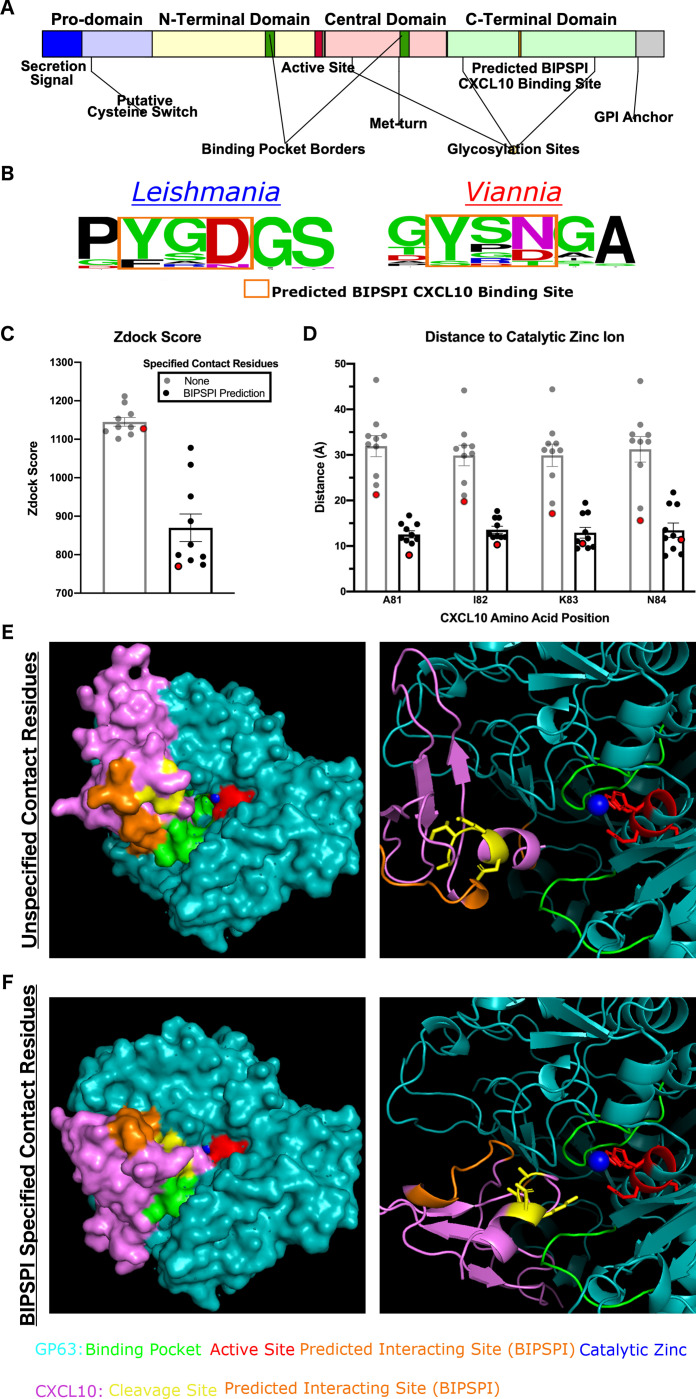
Protein-protein interaction modeling reveals a putative CXCL10 binding site on GP63 that closely approximates the GP63 active site and CXCL10 cleavage site. *(A) The predicted CXCL10 binding site is distal from previously described functional residues on GP63*. Domain diagram of GP63 protein summarizing known functional and structural features. *(B) The predicted CXCL10 binding site is nearly invariant among the Leishmania subgenus at position D463 and has been mutated to N463 preferentially in the Viannia subgenera*. 54 homologues of *gp63* from *L*. *major* (*LmjF_10*.*0460*) were identified by BlastP on TriTrypDB. The sequences represent the *Leishmania* (34), *Viannia* (18), *and Sauroleishmania* (2) subgenera. Multisequence alignment created using ClustalOmega. Sequence logo is shown from AA position 460–465 on the *L*. *major 10*.*0460* sequence. (C-F) *Modeling of the GP63-CXCL10 interaction with specification of the predicted binding site localizes CXCL10 to the active site and decreases distance to catalytic zinc-ion*. GP63 (PDB entry 1lml) and CXCL10 (PDB entry 1o7y) protein-protein interaction was modeled using Zdock with either no specified contact residues or the BIPSPI predicted binding site specified as contact residues. (C) The mean Zdock score, an energy-based scoring function, is plotted for the top 10 model predictions for each condition. (D) The distance from the known cleavage site on CXCL10, in between A81 and I82, to the catalytic zinc ion was measured using PyMol for the top 10 predicted models. Mean distance in angstroms with SEM is plotted. For (C) and (D) the model with the shortest distance between cleavage site and active site is highlighted in red and the model crystal structures shown in (E) and (F) by space-filling and ribbon diagram. GP63 is shown in teal and CXCL10 in purple along with annotation of functional residues as follows: GP63 active site in red, CXCL10 cleavage site in yellow, GP63 binding pocket in green, catalytic zinc ion in blue, and BIPSPI predicted binding sites in orange.

We hypothesized that *gp63* sequence diversity drives the phenotype of reduced CXCL10 cleavage by *L*. *(V*.*) panamensis*. To test this hypothesis, we modeled where CXCL10 binds to GP63 and compared this relative to known functional residues on the protease. The machine learning approach in xgBoost Interface Prediction of Specific-Partner Interactions (BIPSPI) [36] identified a region of three consecutive amino acids on GP63 (Y461, S462, and D463) in the C-terminal domain distant from the predicted matrix metalloprotease substrate binding pocket [37] (Fig 2A and complete results summarized in S1 Data). The site is also distal from known glycosylation sites involved in protein folding and stability [38], the protease active site [39], secretion signal [40], and putative cysteine switch regulatory element [39] (Fig 2A). Further, this site varied between *Leishmania* and *Viannia* subgenera based on 54 available full-length *gp63* sequences (Fig 2B; 34 *Leishmania*, 18 *Viannia*, 2 *Sauroleishmania*). For *L*. *(L*.*) major* and *L*. *(V*.*) panamensis*, which have different capacity to cleave CXCL10 (Fig 1), the multisequence alignment (S1 Fig) shows that there are no shared alleles at amino acid position 463. Thus, the region identified by BIPSI is distinct from known functional domains of GP63 and is divergent between *Leishmania* and *Viannia* subgenera.

We next used *in silico* modeling of the 3D protein-protein interaction using Zdock [41] to test whether this putative CXCL10 binding site results in physical approximation of the GP63 active site to the CXCL10 cleavage site. We previously showed that CXCL10 cleavage occurs with a first single cleavage event by total protein stain, and subsequently identified the cleavage location at the base of the C-terminal α-helix by mass spectrometry [23]. Using this information, we tested the validity of the protein-protein interaction model by measuring the proximity between the GP63 active site catalytic zinc ion and the CXCL10 cleavage site (Fig 2C, 2D, 2E, and 2F). First, GP63 and CXCL10 were loaded into Zdock with no prior information regarding binding. In the top 10 modelling predictions from this unbiased approach, CXCL10 consistently localized to the binding pocket; however, no consensus emerged for the chemokine orientation within the binding pocket and the average distance from cleavage site to catalytic zinc ion was 31.92 ± 7.27 Å (Fig 2D). Second, the Zdock modeling was repeated with the contact residues predicted by BIPSPI specified. This resulted in a consistent fit of CXCL10 into the GP63 binding pocket with the average distance between the CXCL10 cleavage site[23] and catalytic zinc ion decreased to 12.55 ± 2.52 Å (Fig 2D). Notably, the smallest predicted distance between substrate cleavage site and the enzyme catalytic zinc ion was 8.00 Å which is consistent with the observed distance from the co-crystal structure of matrix-metalloprotease-1 and collagen (PDB: 4AUO) [42]. Therefore, the predicted binding site leads to orientation of CXCL10 such that the cleavage site is accessible by the GP63 active site.

### Episodic positive selection occurred at residues surrounding the CXCL10 binding site on GP63

**Fig 3:**
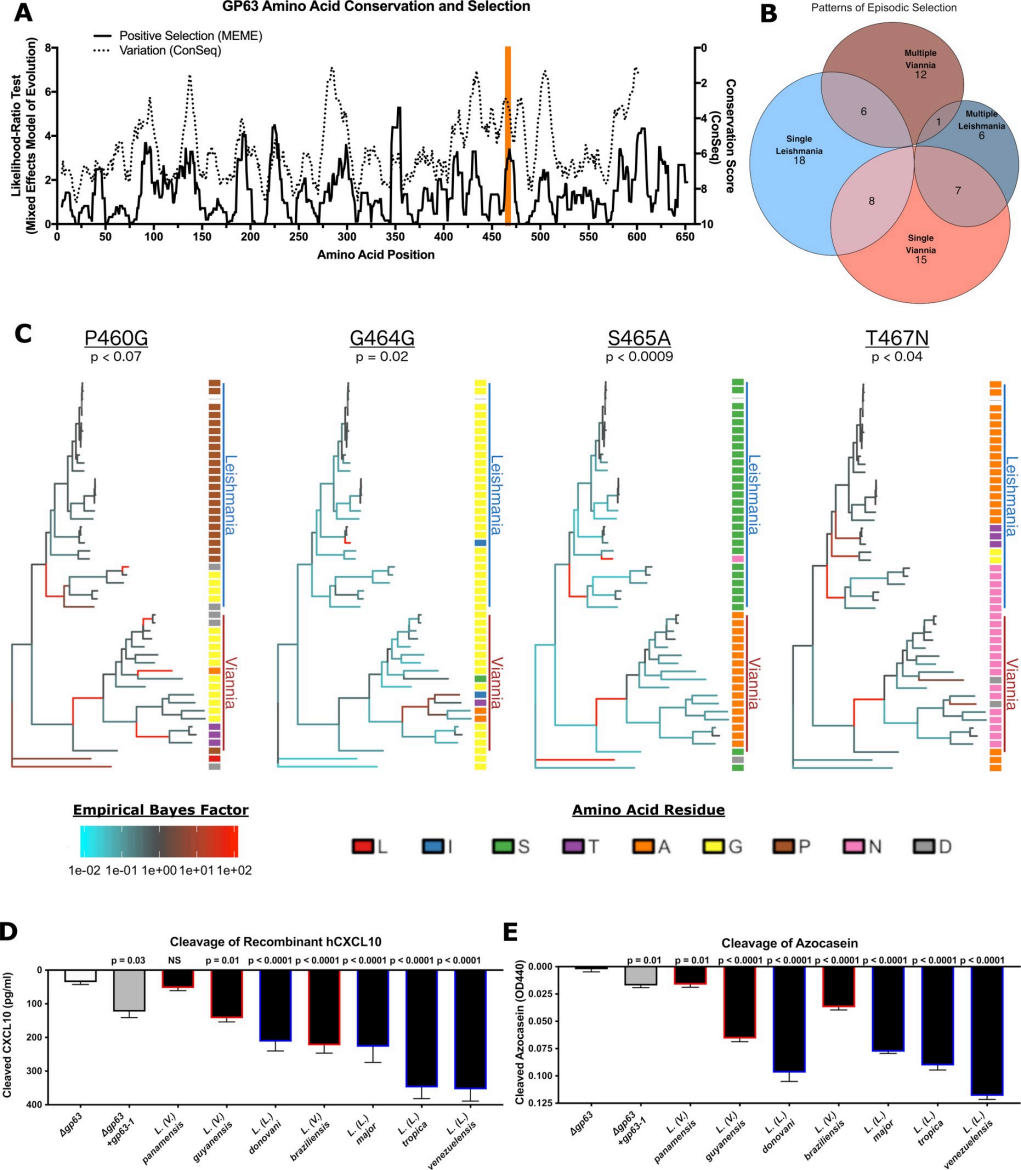
*gp63* has undergone significant positive selection between the *Leishmania* and *Viannia* subgenera, with a peak of selection identified around the CXCL10 binding site. *(A) GP63 contains multiple regions of high diversity under strong positive selection*, *including the region containing the CXCL10 binding site*. The conservation score was generated by the ConSeq method and ranges from 1 (highly variable) to 9 (highly conserved). Positive selection was tested at each amino acid by the likelihood ratio test from the Mixed Effects Model of Evolution (MEME). Higher likelihood ratio indicates stronger signal of positive selection. Both the conservation score and likelihood ratio test are plotted as the moving average over 10 amino acid windows. The putative CXCL10 binding site identified by BIPSPI is highlighted in orange. *(B) GP63 individual amino acids demonstrate diverse patterns of episodic selection*. The MEME test for positive selection generates an empirical bayes factor (EBF) as an estimate of the strength of positive selection for each amino acid along each branch of the phylogenetic tree. Using this measure, the patterns of episodic selection were classified as single or multiple events occurring in either the *Leishmania* or *Viannia* subgenera. EBF was set to a threshold of 30 as a cutoff for positive selection. *(C) Residues adjacent to the CXCL10 binding site demonstrate a pattern of exclusive mutation between the Leishmania and Viannia subgenera*. The EBF generated by MEME was plotted onto the phylogenetic tree for positively selected residues (at a threshold of p<0.1) within 5 amino acids of the predicted CXCL10 binding site. The phylogenetic tree was rooted at the node of the most recent common ancestor of the two *Sauroleishmania* sequences identified. The amino acid residue for each sequence at the indicated position is plotted based on a multisequence alignment generated in ClustalOmega and used for both evolutionary tests above. The *Leishmania* subgenus is labelled with a blue vertical bar, and the *Viannia* subgenus is labelled with a red vertical bar. MEME excludes identical sequences; therefore only 49 of 54 identified sequences are displayed in the phylogeny. P-values calculated based on likelihood ratio test statistic (LRT) as described in [45]. For phylogenies labeled with species and gene name see S2 Fig. *(D)* L. Viannia guyanensis *and* L. Viannia braziliensis *parasites have an intermediate CXCL10 cleavage phenotype consistent with the observation of mixed D463 and Y463 alleles in the* Viannia *subgenus*. The CXCL10 cleavage assay described in Fig 1 was repeated with *L*. *(L*.*) donovani*, *L*. *(L*.*) venezuelensis*, *L*. *(L*.*) tropica*, *L*. *(V*.*) guyanensis*, and *L*. *(V*.*) braziliensis*. 1x10^6^ parasites were incubated with 500 pg/mL of human recombinant CXCL10 at 37°C for 1 hour prior to measuring the remaining CXCL10 by ELISA. Cleaved CXCL10 was calculated by subtracting the CXCL10 in the parasite conditions from a no-parasite media control. Mean ± SEM is plotted from 10 biological replicates across 5 separate experiments. *(E)* Viannia *and* Leishmania *subgenera parasites have comparable general protease activity determined by cleavage of a non-specific substrate*. The azocasein cleavage assay described in Fig 1 was repeated with *L*. *(L*.*) donovani*, *L*. *(L*.*) venezuelensis*, *L*. *(L*.*) tropica*, *L*. *(V*.*) guyanensis*, and *L*. *(V*.*) braziliensis*. 5x10^7^ parasites were incubated with 50 mg/mL of azocasein at 37°C for 5 hours. Cleaved azocasein was determined by subtracting OD440 from the no-parasite media control. Mean OD440 of cleaved azocasein +/- SEM is plotted from 7 biological replicates across 5 independent experiments. For (D) and (E), parasites on the x-axis are ranked by total amount of CXCL10 cleaved, and P-values calculated by one-way ANOVA with Holm-Sidak test comparing each species to the *L*. *major Δgp63* control.

Given the variation between *Leishmania* and *Viannia* parasites (Fig 2B) at the predicted CXCL10 binding site, we hypothesized that this region is under positive selection that potentially contributes to the phenotype of reduced CXCL10 cleavage. Previous studies have described high rates of variation and evidence of positive selection around the protease active site in the tertiary structure [33,34]. However, these studies included 6 or fewer total *Viannia* sequences and only tested for pervasive selection. In the case of *Leishmania* spp. where there is significant heterogeneity within the phylogenetic structure, this may underestimate the degree of positive selection by discounting instances where selection only occurred in a subset of the phylogeny. Therefore, using the 54 sequences identified by BlastP above, we analyzed the evolutionary pressure on *gp63* using two models: 1) the ConSurf model which generates a conservation score based on a maximum likelihood estimate of the evolutionary rate at each amino acid site [43,44] and 2) the Mixed Effects Model of Evolution (MEME) on the HyPhy platform which tests for episodic positive selection at each amino acid residue [45]. The conservation score generated by ConSurf showed extremely high conservation around known functional residues such as the active site, met-turn, and GPI-anchor (Fig 3A). Amino acids with low conservation scores, which are therefore highly variable, correlate with the amino acids identified as under positive selection by MEME (Fig 3A). One such peak of variation and positive selection encompasses the putative CXCL10 binding site identified here (Fig 3A). The high variability and evidence of positive selection around the CXCL10 binding site is consistent with this region being involved in variation in substrate binding.

Next, we asked whether this pattern of amino acid substitution corresponds to a difference in *L*. *(V*.*) panamensis* specifically or as part of a larger subset of parasites such as the *Viannia* subgenus. To do so we utilized the empirical bayes factor (EBF), a measure of the strength of positive selection generated by MEME, to examine the patterns of episodic selection within the phylogeny for each amino acid residue. The 82 amino acid sites identified as under positive selection (P < 0.05) were categorized based on whether they were positively selected along one or multiple branches within either the *Leishmania* or *Viannia* subgenera (Fig 3B). 4 positively selected residues (at a threshold of p<0.1) were identified within 5 amino acid residues of the putative CXCL10 binding site. Three of these sites were mutated away from the consensus *Leishmania* subgenus residue in all sequences from the *L*. *(V*.*) panamensis* reference genome and in at least 17 out of 18 *Viannia* sequences analyzed: P460G, S465A, T467N (Figs 3C and S2). These results suggest that the change in GP63 substrate specificity is generalizable to other parasites in the *Viannia* subgenus.

### Viannia parasites demonstrate variable CXCL10 suppression corresponding to frequency of the CXCL10 specific gp63 allele

To test whether this predicted difference in substrate specificity applied to additional *Leishmania* and *Viannia* parasites, we repeated the CXCL10 cleavage assay using *L*. *(L*.*) donovani*, *L*. *(L*.*) venezuelensis*, *L*. *(L*.*) tropica*, *L*. *(V*.*) guyanensis*, and *L*. *(V*.*) braziliensis*. Similar to *L*. *(V) panamensis*, the other two *Viannia* species tested cleaved less CXCL10 than all *Leishmania* species except for *L*. *(L*.*) donovani* (Fig 3D). Notably, they demonstrated an intermediate capacity to cleave CXCL10, which is consistent with the observation that unlike *L*. *(V*.*) panamensis*, *L*. *(V*.*) braziliensis* parasites have a mixture of *gp63* copies, some of which retain the D463 allele present in the *Leishmania* subgenera while others have the *Viannia* specific N463 allele (Figs 3C and S2). However, each of the *Viannia* species retained some ability to cleave the non-specific azocasein substrate (Fig 3E), indicating that the pattern of decreased GP63 activity in *Viannia* parasites is substrate specific. Together this data suggests that *Viannia* parasite infection results in increased CXCL10 accumulation due to reduced GP63-mediated chemokine suppression in addition to previously described differences in host-parasite sensing.

### *Mutagenesis of positively selected residues near the CXCL10 binding site on* L. (L.) major gp63 to the L. (V.) panamensis sequence significantly impairs CXCL10 cleavage

**Fig 4:**
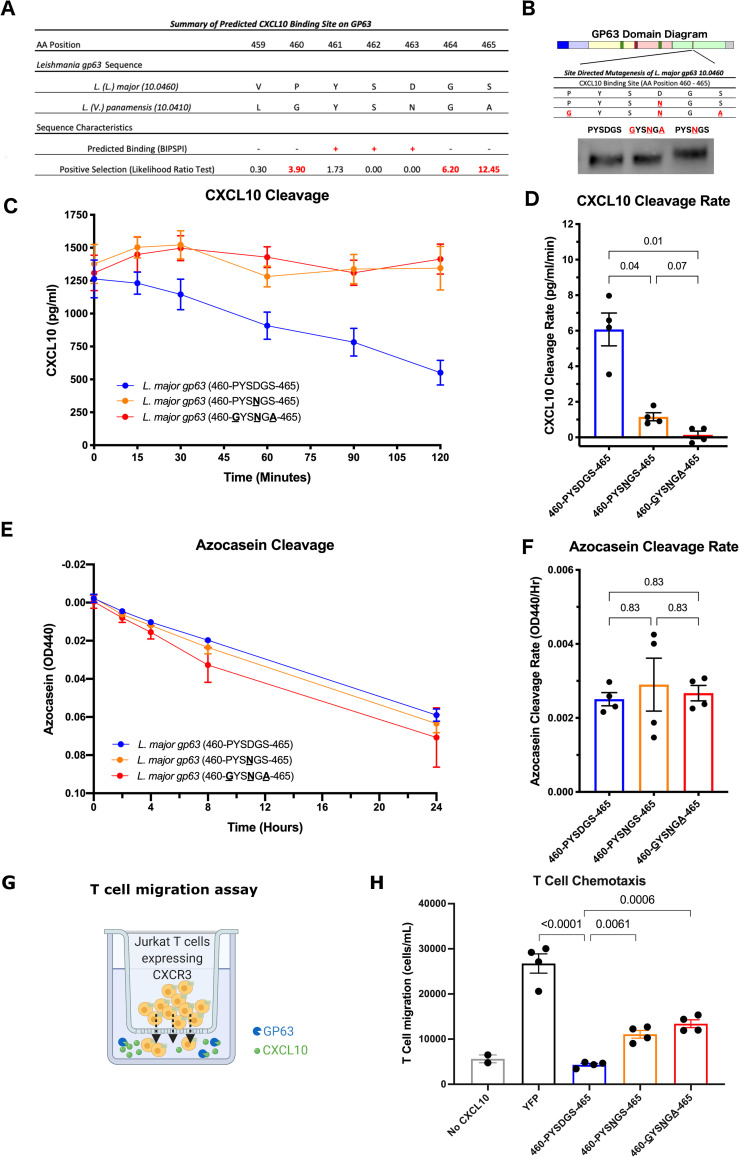
Mutagenesis of the CXCL10 binding site on *L*. *(L*.*) major* GP63 to *L*. *(V*.*) panamensis* residues reduces CXCL10 cleavage and T cell chemotaxis. *(A) The predicted CXCL10 binding site is in a highly variable region of gp63 with multiple adjacent residues under positive selection*. Summary of the predicted *L*. *(L*.*) major* and *L*. *(V*.*) panamensis* CXCL10 binding alleles from BIPSPI analysis and degree of positive selection as measured by likelihood ratio test from MEME. Amino acid position number is reported relative to the *L*. *major gp63 10*.*0460* gene. (B) *Generation of CXCL10 binding site mutants by site-directed mutagenesis of the* L. major gp63 WT *sequence*. The wild-type *L*. *(L*.*) major gp63* CXCL10 binding allele (460-PYSDGS-465) cloned in an overexpression vector was mutated to the *L*. *(V*.*) panamensis* allele at the BIPSPI predicted binding site D463 (460-PYS**N**GS) and at the adjacent positively selected residues (**G**YS**N**G**A**). All three *gp63* plasmids were overexpressed in HEK293T cells and GP63 protein recovered in culture supernatants. Total GP63 protein was assessed by western blot, and samples were then diluted to equal concentration before downstream use. Representative western blot of normalized GP63 from culture supernatants is shown. (C-D) *Mutation of the* L. (L.) major *gp63 allele (D463) to the* L. (V.) panamensis *allele (N463) at the CXCL10 binding site impairs CXCL10 cleavage*. Equal amounts of GP63 protein from each mutant were incubated with 1000 pg/mL of CXCL10 for 2 hours. The remaining CXCL10 in the reaction was measured by ELISA. Change in CXCL10 over time is shown in (C) and the rate of cleavage determined by linear regression is shown in (D). (E-F) *Mutation of the* L. (L.) major gp63 *allele (D463) to the* L. (V.) panamensis *allele (N463) at the CXCL10 binding site does not alter azocasein cleavage*. Equal amounts of GP63 protein from each mutant were incubated with 50 mg/mL of azocasein for 24 hours. Cleaved azocasein was determined by measuring OD440 above background determined by a no transfection media control. Change in azocasein over time is shown in (E) and the rate of cleavage determined by linear regression is shown in (F). For (C-F) mean +/- SEM is plotted from 4 unique experiments representing recombinant GP63 generated from two separate transfections. For D and F, P-values calculated by one-way ANOVA with Holm-Sidak post-hoc test. (G) *Schematic of T cell chemotaxis assay*. 25 ng/mL of CXCL10 was incubated with recombinant GP63 from a single transfection for 20 hours and added to the basal chamber of a transwell. CXCR3^+^ T cells were added to the apical chamber of the transwell. T cell migration was assayed at 2 hours. Image created using Biorender.com. (H) *Mutation of the* L. (L.) major gp63 *allele (D463) to the* L. (V.) panamensis *allele (N463) at the CXCL10 binding site decreases CXCR3*^*+*^ *T cell chemotaxis*. Number of migrated, living T cells is expressed as cells/mL as measured by flow cytometry. Mean ± SEM is plotted from 4 biological replicates across 2 separate experiments. P-values calculated by one-way ANOVA with Dunnett’s multiple comparison test.

We sought to experimentally test how the putative CXCL10 binding site and surrounding residues under positive selection between *Leishmania* and *Viannia* subgenera (summarized in Fig 4A) alter the kinetics of CXCL10 cleavage by GP63. Using site directed mutagenesis of an *L*. *major gp63* overexpression plasmid, we mutated the CXCL10 binding site and nearest positively selected residues (Fig 4B), expressed these constructs from HEK-293T cells, and assayed conditioned media containing equal amounts of WT and mutant GP63. *L*. *major gp63*^*PYSDGS*^, *gp63*^*PYSNGS*^, or *gp63*^*GYSNGA*^ were incubated with CXCL10 at 1000 pg/mL for 2 hours to measure the cleavage rate. The wildtype *gp63*^*PYSDGS*^ had a significantly higher CXCL10 cleavage rate (6.07 pg/mL/min) than both *gp63*^*PYSNGS*^ (1.16 pg/mL/min; p = 0.04) and *gp63*^*GYSNGA*^ (0.14 pg/mL/min; p = 0.01). Notably, the single mutation at position D463 predicted by BIPSPI resulted in incomplete abrogation of CXCL10 cleavage; however, in combination with mutation of the adjacent residues under positive selective pressure the cleavage rate was further reduced (Fig 4C and 4D). To determine whether these mutations caused an overall loss in total proteolytic activity, all copies of *gp63* were also incubated with the non-specific substrate azocasein at 50 mg/mL and monitored over 24 hours. There was no significant difference in azocasein cleavage rate between the wildtype and either mutated copy of *gp63* (Fig 4E and 4F) indicating that the observed loss of CXCL10 cleavage is not due to a reduction in total proteolytic activity. Together these data indicate that sequence variation of GP63 in the *Viannia* subgenus contributes to substrate specificity and differences in chemokine suppression. Specific changes that have been selected for in *L*. *(V*.*) panamensis* and other *Viannia* parasites markedly reduce CXCL10 cleavage while having minimal effect on overall proteolytic activity.

### *The* gp63 *D463N mutation decreases CXCL10 cleavage and results in greater T cell chemotaxis*

As the *gp63* D463N mutation demonstrated reduced cleavage of CXCL10 protein (Fig 4C and 4D), we asked whether this impacts the cellular phenotype of T cell migration. To do so we incubated human recombinant CXCL10 at 25 ng/mL with either *gp63*^*PYSDGS*^, *gp63*^*PYSNGS*^, or *gp63*^*GYSNGA*^ for 20 hours prior to assaying its chemotactic activity across a transwell insert. Jurkat T cells engineered to overexpress CXCR3 [23] were placed in the apical membrane and allowed to migrate towards the CXCL10-GP63 reactions for 2 hours (Fig 4G). *L*. *(L*.*) major* wildtype *gp63*^*PYSDG*^ completely reduced chemotaxis to background levels (Fig 4H). In contrast, consistent with the observed loss of proteolytic activity for CXCL10 by the *gp63*^*PYSNGS*^ and *gp63*^*GYSNGA*^ mutants (Fig 4D), there was significantly greater chemotaxis when CXCL10 was incubated with either *gp63*^*PYSNGS*^ (p = 0.0006) or *gp63*^*GYSNGA*^ (p = 0.00006) (Fig 4H). The longer preincubation time of CXCL10 and GP63 conditioned media (20 hours) compared to the CXCL10 cleavage assays (2 hours in Fig 4C) may have contributed to the reduced chemotaxis relative to incubation with YFP-conditioned media. Nevertheless, our results are consistent with a molecular mechanism of reduced CXCL10 cleavage by *L*. *(V*.*) panamensis* contributing to greater T cell migration, which may result in increased inflammation and cytotoxicity during infection.

### *Within the Viannia subgenus*, *clinical isolates of* L. (V.) braziliensis *have a higher proportion of the CXCL10 cleaving GP63 D463 allele compared to* L. (V.) panamensis

**Fig 5:**
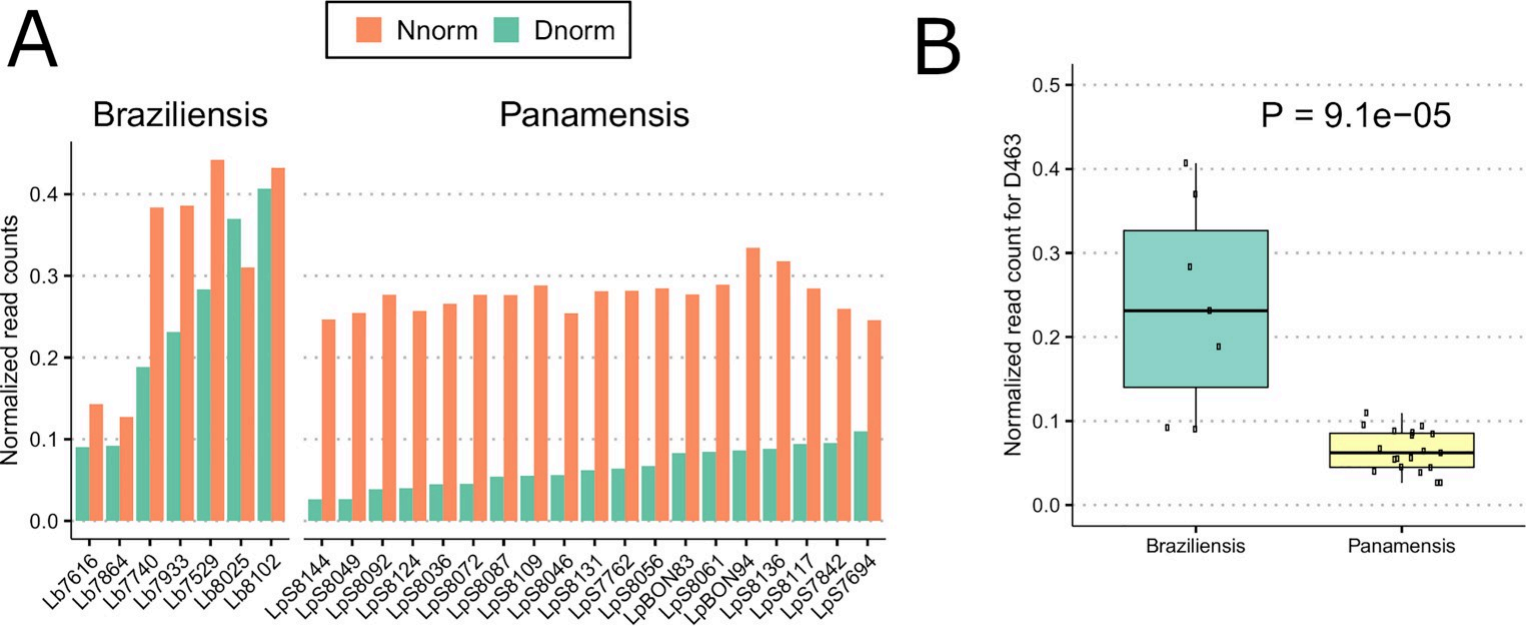
Clinical isolates of *L*. *(V*.*) panamensis* have predominantly CXCL10 non-cleaving N463 allele, whereas *L*. *(V*.*) braziliensis* have the N463 allele in addition to the CXCL10 cleaving D463. *(A) Barplot of proportion of CXCL10 cleaving (D463) and non-cleaving (N463) alleles in individual clinical isolates*. Short read sequences from 7 *L*. *(V*.*) braziliensis* and 19 *L*. *(V*.*) panamensis* were obtained from Patino et al. (2020) and Patino et al. (2020) and aligned to the *L*. *(V*.*) panamensis* PSC-1 reference genome. We then quantified the number of reads carrying the amino acid allele (N, D, A, or T) at *gp63* position 463. The read number was further normalized to total read depth of each sample with a scale factor of 10000, depicted as “Normalized read counts” (y axis). Amino acid position number is reported relative to the *L*. *(L*.*) major 10*.*0460* sequence. (B) L. (V.) braziliensis *clinical isolates have significantly higher proportion of CXCL10-cleaving D463 allele than L*. *(V*.*) panamensis* isolates. Boxplot of the 26 clinical isolates demonstrates the distribution of D463 frequency between *L*. *(V*.*) braziliensis* and *L*. *(V*.*) panamensis* isolates. The normalized read counts (y axis) were calculated as described in Fig 5A. P-value calculated using Wilcoxon rank-sum test.

We next sought to determine the potential for the relative amounts of D463 and N463 alleles to contribute to clinical variation in leishmaniasis by analyzing GP63 sequence variation from recently isolated parasites from Colombia and Bolivia [46,47]. Patino et al. sequenced whole genomes from parasites (19 *L*. *(V*.*) panamensis* [46] and 7 *L*. *(V*.*) braziliensis* [47]) from 26 infected patients. Due to the high rates of *gp63* gene duplication and copy number variation, significant genetic heterogeneity occurs among parasite isolates and the structure of the region is poorly understood, even in the reference PSC-1 strain. Therefore, we aligned the available sequences to the *L*. *(V*.*) panamensis* PSC-1 reference genome [25] and quantified the relative D463 and N463 allele frequencies based on the read depth at all mapped *gp63* positions combined. Notably, all *L*. *(V*.*) panamensis* isolates predominantly encode the N463 allele, which has substantially reduced CXCL10 cleavage activity. However, the *L*. *(V*.*) braziliensis* clinical isolates have greater frequency of the CXCL10 cleaving D463 allele (Fig 5A and 5B). There were also additional less frequent variants that encode substitutions to alanine and threonine at this locus. The variation matched the phylogenetic analysis of *Leishmania* reference sequences (Fig 3C) and was consistent with the intermediate CXCL10 cleavage phenotype observed with *L*. *(V*.*) braziliensis* (Fig 3D). Next, we tested whether the proportion of the CXCL10 cleaving D463 allele was associated with either of the clinical phenotypes reported by Patino et al. [46,47]: number of lesions and type of lesions (S3 Fig). The association with number of lesions, a potential proxy for parasite metastasis, trended towards significance (p = 0.14); whereas there was no association with the type of lesion observed (p = 0.97). However, with only 26 patient samples this cohort was ultimately underpowered to detect a statistical association between *gp63* genotype and clinical outcome. The observation that both the CXCL10 cleaving and non-cleaving allele are present in naturally occurring *Viannia* isolates confirmed the relevance of this diversity in clinical isolates and suggests that human infection likely also varies in the type and robustness of CXCL10 mediated inflammation during infection.

## Discussion

Genetic diversity of *Leishmania* spp. contributes to variable disease presentation, but how the diversity alters molecular mechanisms to impact disease outcome is not completely understood. Here we described how variation in chemokine suppression by the virulence factor GP63 between the *Leishmania* and *Viannia* subgenera is driven by genetic variation generated by positive selection. Thus, we have identified the molecular and evolutionary basis for one difference in immune evasion between closely related parasites, and undoubtedly more remain to be discovered.

We characterized a change in GP63 substrate specificity for human CXCL10; however, the genetic variation between subgenera potentially confers loss or gain of specificity for other GP63 substrates. GP63 has a myriad of described substrates [20] with roles in host defense against infection ranging from interfering with complement mediated lysis [21] to altering intracellular signaling [48–50] to impairing antigen cross-presentation [51]. Our work here shows that cross species variation significantly alters GP63 function, resulting in a change in chemokine landscape during infection. Future studies are needed to establish whether *Leishmania* diversity influences GP63 specificity and activity for other known substrates. In particular, GP63 substrates known to inhibit cytokine release, such as Synaptotagmin XI [52], should be examined to determine the net effect on chemokine response during infection. It is also possible that mutations have led to gain of function for currently undiscovered substrates, which may alter chemokine signaling or other aspects of the immune response that contribute to the diverse clinical outcomes associated with *Leishmania* infection. Ultimately, to connect this novel molecular mechanism to organismal phenotypes, future studies should overexpress alternative GP63 allele’s in *Leishmania* and *Viannia* parasites during *in vitro* and *in vivo* infection.

Variation in chemokine response due to GP63 diversity is likely to contribute to differences in severity of disease outcome by altering the balance between a well-regulated protective host immune response and dysregulated immune mediated pathology. *Leishmania major* was used as a model organism to establish the paradigm of a protective T_h_1 cell response against intracellular pathogens [5,6]. However, recent advances have described that severe, ulcerative lesions caused by *Viannia* subgenera parasites, including *L*. *(V*.*) panamensis* and *L*. *(V*.*) braziliensis*, are characterized by markedly increased expression of type-1 associated cytokines and chemokines including CXCL10 [13,53]. This is known to be in part due to differences in host Toll-like receptor recognition of the parasite leading to differences in transcriptional changes [15,27]. Here we uncovered another layer of this complex host-pathogen interaction where in addition to differences in host sensing, *Viannia* parasites appear to rely on alternative mechanisms of immune evasion to the related *Leishmania* parasites. While we observed that both CXCL10 cleaving and non-cleaving alleles occurred in *L*. *(V*.*) panamensis* and *L*. *(V*.*) braziliensis* clinical isolates, the sample size and limited clinical information was insufficient to determine if the *gp63* allele was associated with specific clinical outcomes. Thus, future studies are needed to test how this diversity in parasite manipulation of chemokine signaling impacts pathogenesis in animal models and human disease. Specifically, given the significant *gp63* genetic diversity in both nucleotide and copy number variations, long-read sequencing from a large number of clinical derived samples is warranted to accurately map this region and test for associations with clinical outcomes.

The observation that genetic diversity contributes to variation in immune evasion raises the question as to what factors are responsible for generating and maintaining greater diversity in the *Viannia* subgenus. The diversification appears to be facilitated by the large copy number expansion in the *Viannia* subgenus [28,54]. It is possible that the initial gene expansion of *gp63* was driven by pressure to counteract host CXCL10 production increased in New World *Viannia* infections in part due to the presence of RNA viruses infecting either parasite or host [12–15,17]. Consistent with a model of environmental pressure in the New World driving the expansion of GP63, Bussotti et al. have described a large copy number expansion of GP63 in an *L*. *(L*.*) infantum* isolate from Brazil compared to isolates from the Old World [55]. Such gene duplications are a common mechanism allowing for diversification of novel genes in multiple biological systems [56]. By maintaining the original copy, the novel copy confers freedom to explore novel biochemical space and test new strategies for parasite survival, persistence, and spread. Eventually with a different strategy to survive in mammalian hosts, the dependence on the older mechanism of CXCL10 suppression could become expendable leading to loss of the CXCL10 specific alleles. Further, the greater diversity of *gp63* maintained within the *Viannia* subgenera may itself provide an evolutionary advantage. Within 41 *L*. *(V*.*) braziliensis* isolates from a single location in Brazil, 45 different polymorphic alleles were identified within *gp63* [29], and *L*. *(V*.*) braziliensis* isolates vary significantly in several phenotypes of immune modulation *in vitro* [32]. A larger pool of sequences conferring unique substrate specificities can allow for rapid adaptation to new environmental or host challenges. Additional studies are warranted to further characterize the evolutionary and functional implications of *gp63* diversity within the *Viannia* subgenus. Regardless of how the expansion and diversification occurred, our results clearly demonstrate that current *Leishmania* isolates have genetic variation in *gp63* that contributes to differences in host chemokine levels during infection. Further, degree of CXCL10 suppression appears to have been selected for by the particular niche occupied by each *Leishmania* species, where variability in the host environment results in balancing selection and maintenance of diversity for the *Viannia* subgenus.

*Leishmania* genetic diversity creates a complex and challenging set of interactions between host and parasite but also represents a significant opportunity to leverage that diversity to improve our understanding of mechanisms of pathophysiology. Studies such as this one that link genetic variation to phenotypic differences in chemokine signaling will be required to fully understand the parasite factors that contribute to differential host susceptibility to infection. A more complete understanding of this molecular evolution will facilitate the development of biomarkers and host-directed therapies to improve outcomes of leishmaniasis.

## Materials and methods

### Human cell lines and culture

THP-1 monocytes, originally from the American Type Culture Collection (ATCC, USA), were obtained from the Duke Cell Culture Facility and maintained in RPMI 1640 media (Invitrogen, USA) supplemented with 10% fetal bovine serum (FBS), 2 mM glutamine, 100 U/mL penicillin-G, and 100 mg/mL streptomycin. HEK293T cells were obtained from ATCC and maintained in DMEM complete media (Invitrogen, USA) supplemented with 10% FBS, 100 U/mL penicillin-G, and 100 mg/mL streptomycin. All cell lines were maintained at 37°C with 5% CO_2_. For phorbol 12-myristate 13-acetate (PMA) differentiation of THP-1 monocytes, 1.2 × 10^6^ cells were placed in 2 mL of complete RPMI 1640 media supplemented with 100 ng/mL of PMA for 16 hours after which the RPMI media was replaced and cells allowed to rest for 24 hours prior to infection.

### Parasite culture and infections

All *Leishmania* parasites were maintained in M199 media supplemented with 100 U/mL penicillin/streptomycin and 0.05% hemin. The following parasites strains were obtained from Biodefense and Emerging Infections (BEI) Resources or ATCC: *L*. *major* Δ*gp63* [(MHOM/SN/74/SD) Δ*gp63 1–7*, NR-42489], *L*. *major* Δ*gp63*+*1* [(MHOM/SN/74/SD) Δ*gp63 1–7* + *gp63-1*, NR-42490], *L*. *major* Friedlin V1 [(MHOM/IL/80/FN) NR-48815], *L*. *tropica* [(MHOM/AF/87/RUP) NR-48820], *L*. *donovani* [(MHOM/SD/62/1S) NR-48821], *L*. *venezuelensis* [(MHOM/VE/80/H-16) NR-29184], *L*. *braziliensis* [(MHOM/BR/75/M2903) ATCC-50135], *L*. *guyanensis* [(MHOM/BR/75M4147), ATCC-50126], and *L*. *panamensis* [(MHOM/PA/94/PSC-1), NR-50162]. Prior to infection parasites were washed once with Hanks Buffered Salt Solution (HBSS) and counted by hemocytometer prior to resuspending in the indicated assay media.

### Human chemokine detection

Human CXCL10 mRNA and protein were detected after infection *in vitro*. At the time of harvest, infected cells were spun at 200g for 5 minutes and the supernatants removed and stored at -80°C storage for cytokine detection. Cells were resuspended in RNAprotect Cell Reagent (Qiagen, Germany) and stored at -80°C prior to extracting RNA with RNeasy RNA extraction kit (Qiagen, Germany). Reverse transcriptase was performed using iScript Reverse Transcriptase kit (BioRad, USA, 1708840). Quantitative real-time PCR (qRT-PCR) was performed using iTaq Universal Probes Supermix (Biorad, USA, 1725135) with human CXCL10 (ThermoFisher, USA, Hs01124252) or rRNA45s5 (Thermo, USA, Hs03928990_g1) probes. Relative expression was calculated by the ΔΔCt method relative to the rRNA45s5 housekeeping gene. hCXCL10 protein concentration in supernatant was assayed by ELISA (R&D Systems, USA, 266-IP).

### Prediction and modeling of CXCL10 binding site on GP63

*In silico* prediction of the CXCL10 binding site on GP63 involved two sequential steps utilizing the amino sequence and solved crystal structures in the Protein Data Bank (GP63: 1lml and CXCL10: 1o7y). First, the primary amino acid sequences for GP63 and CXCL10 were input to the xgBoost Interface Prediction of Specific-Partner Interactions (BIPSPI) [36] webserver (http://bipspi.cnb.csic.es/xgbPredApp/)) to predict the interacting residues. Second, the crystal structures for GP63 and CXCL10 were uploaded to the Zdock webserver (http://zdock.umassmed.edu/) [41] to model the protein-protein docking interaction. The Zdock modeling was performed under two sets of conditions: 1) blinded to any knowledge of predicted contact residues and 2) with the contact residues identified by BIPSPI specified. The top ten models for the GP63-CXCL10 interaction under each conditioned were visualized using PyMol [57]. Distance measurements were performed with the PyMol “distancetoatom” function relative to the catalytic zinc ion in GP63 (PDB 1lml).

### Evolutionary analysis of GP63 sequences

To examine the evolutionary pressure on GP63 we 1) identified a set of GP63 sequences to create a multisequence alignment, 2) tested for the degree of conservation at each amino acid residue, and 3) tested for episodic positive selection at individual amino acid residues. First, GP63 sequences were identified using BlastP with *gp63* from *L*. *major* Fd (*LmjF_10*.*0460*) used as a query (E: 0.01, no low complexity filter) on TriTrypDB [58] to search all *Leishmania* spp. The identified sequences and identifying information were downloaded. The publicly available sequences were then filtered based on the following characteristics: length (greater than half the length of the reference GP63 (302 amino acids), presence of ambiguous amino acids, genomic location (restricted to the chromosome 10 locus of *gp63*). The remaining 54 full length nucleotide sequences from the chromosome 10 locus were then aligned using ClustalOmega [59]. Finally, using AliView [60] the alignment was manually inspected, all stop codons were removed, and the nucleotide sequence was translated to amino acid sequence. Second, the degree of conservation at each position was analyzed using the ConSurf [43] server with the ConSeq [44] method. The translated amino acid multi-sequence alignment created above was uploaded, the 3D structure was not specified in order to include residues from the complete peptide (the crystal structure was only solved beginning at residue 100), setting *LmjF_10*.*0460* as the query sequence, and calculating the phylogenetic tree by Bayesian method. Third, episodic positive selection was tested for by the Mixed Effects Model for Evolution [45] on the Datamonkey 2.0 server [61] using the nucleotide alignment generated above. The empirical bayes factor was mapped onto the generated phylogenetic tree using the ggtree [62] package in R [63]. Prior to mapping the EBF, the phylogenetic tree was rooted to the node at the base of the *Sauroleishmania* subgenus using the root function in the ape package [64].

### Expression of recombinant GP63 and site-directed mutagenesis

Overexpression and mutagenesis of GP63 was performed as described previously [23]. In brief, 250,000 HEK293T cells were washed once with PBS, resuspended in serum free, FreeStyle 293 Expression Media (ThermoFisher, USA, 12338018), and plated in a 6-well tissue culture treated dish 48 hours before transfection. One hour prior to transfection, media was replaced with fresh FreeStyle 293 media. Transfections were performed following the manufacturer’s protocol with the Lipofectamine 3000 transfection reagent kit. Supernatants were harvested 48 hours after transfection and stored in single use aliquots in low binding tubes at -80°C. Site directed mutagenesis was performed using the Agilent (USA) Quick Change Site Directed Mutagenesis kit per the manufacturers protocol.

### CXCL10 and azocasein cleavage assays

To assay GP63 activity two substrates were used: human CXCL10 and the non-specific colorimetric protease substrate azocasein. To normalize the total number of live parasites assayed in each reaction: 8mL of a day 6 culture of promastigotes was washed once with HBSS and counted by hemocytometer to load an equal number of parasites for each substrate reaction. For heterologous expressed GP63, the relative amount of GP63 in each reaction was normalized based on total GP63 detected by western blot for a C-terminal histidine epitope tag. Protein was first separated by electrophoresis in a 4–20% bis-tris polyacrylamide gel before transferring to PVDF membrane using a Hoefer TE77X semi-dry transfer system. GP63 was detected by primary anti-his antibody (Cell Signaling Technology, USA, 12698) at 1:200 dilution with a secondary anti-rabbit fluorescent probe (Licor, USA, IRDye 800CW) at 1:20,000 dilution and developed with LiCor Odyssey Infrared Imaging System. Relative band intensity was quantified and used to produce even loading of GP63 mutants in the subsequent cleavage reaction. Dilutions of the GP63 mutants were rerun by western to confirm that equal amounts of protease were added to all reactions.

To monitor GP63 cleavage of CXCL10, normalized live parasites (1x10^6^) or heterologously expressed GP63 was incubated with 500 pg/mL of human recombinant CXCL10 (Peprotech, USA) for the indicated time at 37°C. To ensure equal input of the different GP63 constructs to each reaction, the relative concentration of heterologously expressed GP63 constructs was determined in duplicate by western blot as described above and normalized within each experiment. The remaining CXCL10 was assayed by hCXCL10 enzyme-linked immunosorbent assay (ELISA) (R&D Systems, USA, 266-IP). The cleaved fraction of CXCL10 was calculated by subtracting the remaining measured CXCL10 from the no parasite or no transfection control. To monitor GP63 cleavage of azocasein cleavage 50 μL of normalized live parasites (5x10^7^) or heterologously expressed GP63 was incubated with 200 μL of 50 mg/mL of azocasein (Sigma-Aldrich, USA, A2765) for the indicated time at 37°C. To stop the reaction 50 μL of the reaction was added to 200 μL of 5% trichloroacetic acid (TCA). The precipitate was spun at 2200g for 10 minutes, and 150 μL of supernatant transferred to a clean well of a clear bottom 96-well plate. Finally, 112.5 μL of 500 mM NaOH was added to each well and absorbance was subsequently measured at OD440 using a BioTek (USA) microplate reader. The cleaved fraction of azocasein was calculated relative to the no parasite or no transfection control.

### Quantification of gp63 expression

To quantify *gp63* expression, mRNA was obtained from day 6 of promastigote cultures of either *L*. *(L*.*) major* or *L*. *(V*.*) panamensis*. Parasites were washed once with HBSS and counted by hemocytometer prior to resuspending at a concentration of 1x10^6^ parasites/60 μL and incubating at 37°C for 1 hour to mimic the THP-1 infection conditions. A total of 4 mL (6.67x10^7^ parasites) were used for harvesting RNA. At the end of 1 hour, parasites were spun at 1300g for 10 minutes and resuspended in buffer RLT from the RNeasy Mini Kit (Qiagen, Germany). Genomic DNA was removed from the sample using TurboDNase (ThermoFisher, AM2239) per manufacturers protocol. cDNA synthesis was performed with the iScript Reverse Transcriptase kit (BioRad, USA, 1708840) with 1 μg total RNA input for each sample. cDNA was then used directly and undiluted in downstream qPCR reactions using the iTaq Univeral SYBR Green Mastermix (BioRad, USA, 172–5124) with 50 nm of each primer and 4 μL of cDNA for gene targets or 2 μL of cDNA for housekeeping genes in a final reaction volume of 10 μL. Relative expression was calculated as ΔCt.

Primers for *gp63* were designed to capture all copies in the chromosome 10 locus for *L*. *(L*.*) major* and *L*. *(V*.*) panamensis*. For *L*. *(L*.*) major* there are four copies of *gp63* in a tandem array of which 1 copy is sufficiently divergent from the other three to require unique primers. Therefore, one set of primers were designed to amplify *LmjF_10*.*0460*, *LmjF_10*.*0465*, and *LmjF_10*.*0480* (Fwd: CCGTCACCCGGGCCTT, Rev: CAGCAACGAAGCATGTGCC) and a separate set of primers to amplify *LmjF_10*.*0470* (Fwd: TTGAGCGGTGGAATGAGAGG, Rev: AGTGCCATGAGAGAGAGAACT). For *L*. *(V*.*) panamensis* all our copies were homologous enough to utilize one set of primers for all four copies: *LPMP_100410*, *LPMP_100420*, *LPMP_100430*, and *LPMP_100440* (Fwd: CCGACTTCGTGCTGTACGTC, Rev: TGAAGCCGAGGGCGTG). Previously described primers for the α-tubulin housekeeping gene [65] were used as a control because the gene is highly conserved between *L*. *(L*.*) major* and *L*. *(V*.*) panamensis*.

### Whole genome assembly and analysis of gp63 substrate specific alleles

To quantify the natural diversity of GP63 sites involved in CXCL10 substrate specificity, we reassembled the whole genomes of 26 samples from *Viannia* subgenus parasites, including 19 *L*. *(V*.*) panamensis* and 7 *L*. *(V*.*) braziliensis* parasites. Raw data (.sra) were downloaded from two previous studies [46,47]. The module *fastq-dump* from NCBI SRA toolkit v2.10.9 (https://github.com/ncbi/sra-tools/) was used to convert the raw data to FASTQ format. BWA-MEM v0.1.17[66] was then used to align all short reads to the *L*. *(V*.*) panamensis* PSC-1 reference genome. The reference genome was downloaded from TriTryDB database (available at https://tritrypdb.org/tritrypdb/app/record/dataset/DS_21a844223f). On this reference genome, there are four *gp63* copies (gene names: LPMP_100410, LPMP_100420, LPMP_100430, LPMP_100440) on chromosome 10, and one distantly related *gp63*-like protein (LPMP_311850) on chromosome 31 which was not included in this study.

Sequence alignment to the reference genome was performed with minimum seed length of exact match as 19 (-k). Duplicated reads were marked and filtered by *Picard* using the *MarkDuplicates* function (*Picard* was downloaded from http://broadinstitute.github.io/picard). SAMTOOLs v1.9 [67] was then used to sort and index BAM files, and the module BCFTOOLs used to pileup, count read depth, and call variants with quality filtering score of 30 (-q = 30). The final variants were stored into a VCF file. Since the alleles of a variant can have different representations, we used *vt* [68] to normalize variants, and then removed duplicate variants to avoid potential inconsistent calling bias. Next, we counted the read depth mapped to GP63 amino acid position 463. All amino acid numbering for *gp63* described here is reported relative to the *L*. *(L*.*) major* gene *10*.*0460*. A total of four types of amino acid codons were observed: N (AAT, AAC), D (GAT, GAC), T (ACT) and A (GCT). Then we counted the number of reads mapped to each codon for each sample. To account for differences in the sequence library size, the final values were normalized by total library size and multiplied by a scale factor of 10000.

### T cell chemotaxis assays

To quantify if the *gp63* D463N mutation reduced T cell migration, we incubated 25 ng/mL of human recombinant CXCL10 with either *gp63*^*PYSDGS*^, *gp63*^*PYSNGS*^, or *gp63*^*GYSNGA*^ for 20 hours at 37°C. The CXCL10 and *gp63* reaction mixture was added to the basal chamber of a transwell insert (VWR, USA, 10769–236). 250,000 Jurkat T cells engineered to overexpress CXCR3 [23] were added to the apical chamber of the transwell insert. After 2 hours, the cells migrated into the basal chamber were stained using the 7AAD viability dye and quantified using a Guava EasyCyte HT flow cytometer (Millipore, USA).

### Statistical analysis

All statistical tests were performed with GraphPad Prism and with the R statistical programming environment [69]. Student’s T-test, Wilcoxon rank sum test, and one-way ANOVA with Holm-Sidak post-hoc test were used as indicated in the text. The number of biological replicates (N) is indicated in the figure legend for each experiment. For *in vitro* experiments a biological replicate was defined as unique well of cells or chemokine at the time of experimental manipulation by addition of parasites or recombinant GP63 as indicated. All numerical values used in generating graphs in figures are in S2 Data.

## Supporting information

S1 Data BIPSPI Results. The xgBoost Interface Prediction of Specific-Partner Interaction (BIPSPI) algorithm to predict protein-protein interactions from sequential data was run using the primary amino acid sequence used for the solved crystal structures of CXCL10 (PDB: 1o7y) and GP63 (PDB: 1LML). The algorithm generates both predictions for each protein individually and for the interaction of amino acid residues between the two proteins.
(XLSX)

S2 Data Numerical values used in generating graphs. (XLSX)

S1 Fig Complete multisequence alignment of gp63 homologues. 54 homologues of *gp63* from *L*. *major* (*LmjF_10*.*0460*) were identified by BlastP on TriTrypDB. The sequences represent the *Leishmania* (34), *Viannia* (18), *and Sauroleishmania* (2) subgenera. Multisequence alignment created using ClustalOmega, and visualized using the Boxshade server (https://embnet.vital-it.ch/software/BOX_form.html).
(PDF)

S2 Fig Episodic positive selection around the CXCL10 binding site on GP63 with labelled phylogenies. Residues adjacent to the CXCL10 binding site demonstrate a pattern of exclusive mutation between the Leishmania and Viannia subgenera. The EBF generated by MEME was plotted onto the phylogenetic tree for positively selected residues (at a threshold of p<0.1) within 5 amino acids of the predicted CXCL10 binding site. The phylogenetic tree was rooted at the node of the most recent common ancestor of the two Sauroleishmania sequences identified. The amino acid residue for each sequence at the indicated position is plotted based on a multisequence alignment generated in ClustalOmega and used for both evolutionary tests above. MEME excludes identical sequences; therefore only 49 of 54 identified sequences are displayed in the phylogeny. P-values calculated based on likelihood ratio test statistic (LRT) as described in Murrell et al. 2012 [45]. Tip labels include species and genomic location. For species labels: “LBRM” is *L*. *(V*.*) braziliensis* MHOM/BR/75/M2904, “LDCL” is *L*. *(L*.*) donovani* CL-SL, “LINF” *is L*. *(L*.*) infantum* JPCM5, “LMJF” is *L*. *(L*.*) major* strain Friedlin, “LMXM” is *L*. *(L*.*) mexicana* MHOM/GT/2001/U1103, “LPMP” is *L*. *(V*.*) panamensis* strain MHOM/PA/94/PSC-1, and “LTAP” is *L*. *tarentolae* Parrot-TarII.
(DOCX)

S3 Fig Association of CXCL10 binding D463 allele with clinical phenotypes of lesion number and lesion type. As *gp63* is present in a tandem array with variable copy number, we tested for associations of the proportion of the D463 allele with lesion number (A) or lesion type (B). Clinical characteristics were obtained from Patino et al. (2020) and Patino et al. (2020) [46,47]. Lesion number greater than one was used as a biological proxy for metastatic spread of infection. *L*. *(V*.*) braziliensis* isolates are colored in teal and *L*. *(V*.*) panamensis* isolates are colored in yellow. P-values obtained by two-tailed Mann-Whitney rank order test. To calculate the proportion of D463 allele: short read sequences from 7 *L*. *(V*.*) braziliensis* and 19 *L*. *(V*.*) panamensis* isolates were obtained from Patino et al. (2020) and Patino et al. (2020) [46–47] and aligned to the *L*. *(V*.*) panamensis* PSC-1 reference genome. We then quantified the number of reads carrying the amino acid allele (N, D, A, or T) at *gp63* position 463. The read number was further normalized to total read depth of each sample with a scale factor of 10000. Finally, the number of normalized D reads was divided by the normalized total *gp63* reads to get the proportion of the D463 allele.
(DOCX)
