# Supplementary material for: Variation in Leishmania chemokine suppression driven by diversification of the GP63 virulence factor
Source: PLoS Negl Trop Dis. 2021 Oct 28;15(10):e0009224. doi: 10.1371/journal.pntd.0009224 (PMC8577781; doi:10.1371/journal.pntd.0009224)
Supplement: S1 Fig — 54 homologues of gp63 from L. major (LmjF_10.0460) were identified by BlastP on TriTrypDB. The sequences represent the Leishmania (34), Viannia (18), and Sauroleishmania (2) subgenera. Multisequence alignment created using ClustalOmega, and visualized using the Boxshade server (https://embnet.vital-it.ch/software/BOX_form.html). (PDF) [file pntd.0009224.s003.pdf]

```
LPMP_100440      1 MPLDRSSTHRRRSVAARLMRLAAAGLVMAVGAAX-XVW--AQAAGHHCIHDRLQTRVLQS
LbrM.10.0480     1 MSRDRSSTHRRRSVAARLMRLAAAGLVMAVGAAX-XVW--VQAAGHHCIHDRLQARVLQS
LbrM.10.0570     1 ------------------------------------------------------------
LPMP_100430      1 ---------------------------MAVGAAX-XVW--AQAAGHHCIHDRLQTRVLQS
LbrM.10.0470     1 -------MSRDRSVTARLMRLAAAGLVMAVGAAX-XVW--AQAAGHHCIHDRLQARVLQS
LbrM.10.0520     1 MSRDRSSTHRRRSDAARLMRLAAAGLVMAVGAAX-XVW--AQAAGHHCIHDNLQARVLQS
LbrM.10.0540     1 ------------------MRLAAAGLVMAVGAAX-XVW--AQAAGHHCIHDKLQTRVLQS
LbrM.10.0590     1 MPLDSSSTHRRRSVAARLVRLAAAGVAAALAVGTAAAWAHAAATPHRCIHDKLQARVRDS
LPMP_100420      1 MSRDRSSTHRRRSVAARLMRLAAAGLVMAVGAAX-XVWX-XQAAGHHCIHDRLQTRVLQS
LbrM.10.0530     1 ------------------------------------------------------------
LbrM.10.0600     1 MPLDSSSTHRRRSVAARLVRLAAAGVAAALAVGTAAAWAHAAATPHRCIHDKLQARVRDS
LPMP_100410      1 ---------------------------MAVGAX-XAVWVQX-XAGHHCIHDRLQTRVLQS
LbrM.10.0510     1 MPLDSSSTPRRRSVAARLMRLAAAGLVMAVGAX-XAVWAQX-XAGHHCIHDKLQTRVLQS
LbrM.10.0500     1 ------------------------------------------------------------
LbrM.10.0580     1 MPRDSSSTPRRRSVAARLMRLAAAGLVMAVGAX-XAVWAQX-XAGHHCIHDKLQTRVLQS
LbrM.10.0610     1 MPLDSSSTHRRRSDAARLMRLAAAGLVMAVGAX--XAWAQX-XAGHHCIHDKLQARVLQS
LbrM.10.0550     1 MPLDSSSTHRRRSDAARLMRLAAAGLVMAVGAX--XAWAQX-XAGHHCIHDKLQTRVLQS
LbrM.10.0560     1 MPLDSSSTHRRRSDAARLMRLAAAGLVMAVGAX-XAVWAQX-XAGHHCIHDKLQARVLQS
LtaP10.0480      1 ----------------------------------------------------MQARMRQS
LtaP10.0650      1 ------------------------------------------------------------
LmxM.10.0405     1 MSVDSSSTHRNRCVAARLVRLAAAGAAVTVAVGTAAAWAHAGAPQHRCIHDAMQARVRQS
LmxM.10.0460     1 MPVDSSSTHRHRCVAARLVRLAAAGAAVTVAVGTAAAWAHAGAPQHRCIHDAMQARVLQS
LmxM.10.0465     1 MPVDSSSTHRHRCVAARLVRLAAAGAAVTVAVGTAAAWAHAGAPQHRCIHDAMQARVLQS
LmxM.10.0390     1 MSVDSSSTHRNRCVAARLVRLAAAGAAVTVAVGTAAAWAHAAAPQHRCIHDAMQARVRQS
LmxM.10.0470     1 MPVDSSSTHRHRCVAARLVRLAAAGAAVTVAVGTAAAWAHAGAPQHRCIHDAMQARVRQS
LINF_100011200   1 MSVDSSSTHRHRSVAARLVRLAAAGAAVIAAVGTAAAWAHAGAVQHRCIHDAMQARVRQS
LdCL_100011300   1 MSVDSSSTHRHRSVAARLVRLAAAGAAVIAAVGTAAAWAHAGAVQHRCIHDAMQARVQQS
LmjF.10.0480     1 MSVDSSSTHRRRCVAARLVRLAAAGAAVTVAVGTAAAWAHAGALQHRCVHDAMQARVRQS
LmjF.10.0460     1 MSVDSSSTHRRRCVAARLVRLAAAGAAVTVAVGTAAAWAHAGALQHRCVHDAMQARVRQS
LmjF.10.0465     1 MSVDSSSTHRRRCVAARLVRLAAAGAAVTVAVGTAAAWAHAGALQHRCVHDAMQARVRQS
LmjF.10.0470     1 MSVDSSSTHRRRCVAARLVRLAAAGAAVTVAVGTAAAWAHAGALQHRCVHDAMQARVRQS
LINF_100010100   1 MSVDSSSTHRHRSVAARLVRLAAAGAAVIAAVGTAAAWAHAGAVQHRCIHDAMQARVRQS
LdCL_100010700   1 MSVDSSSTHRHRSVAARLVRLAAAGAAVIAAVGTAAAWAHAGAVQHRCIHDAMQARVRQS
LdCL_100010500   1 MSVDSSSTHRHRSVAARLVRLAAAGAAVIAAVGTAAAWAHAGAVQHRCIHDAMQARVRQS
LdCL_100010100   1 MSVDSSSTHRHRSVAARLVRLAAAGAAVIAAVGTAAAWAHAGAVQHRCIHDAMQARVRQS
LdCL_100010300   1 MSVDSSSTHRHRSVAARLVRLAAAGAAVIAAVGTAAAWAHAGAVQHRCIHDAMQARVRQS
LdCL_100011800   1 MSVDSSSTHRHRSVAARLVRLAAAGAAVIAAVGTAAAWAHAGAVQHRCIHDAMQARVRQS
LINF_100011300   1 MSVDSSSTHRHRSVAARLVRLAAAGAAVIAAVGTAAAWAHAGAVQHRCIHDAMQARVRQS
LdCL_100011400   1 MSVDSSSTHRHRSVAARLVRLAAAGAAVIAAVGTAAAWAHAGAVQHRCIHDAMQARVQQS
LdCL_100010900   1 MSVDSSSTHRHRSVAARLVRLAAAGAAVIAAVGTAAAWAHAGAVQHRCIHDAMQARVRQS
LdCL_100011600   1 MSVDSSSTHRHRSVAARLVRLAAAGAAVIAAVGTAAAWAHAGAVQHRCIHDAMQARVRQS
LdCL_100011200   1 MSVDSSSTHRHRSVAARLVRLAAAGAAVIAAVGTAAAWAHAGAVQHRCIHDAMQARVRQS
LdCL_100011500   1 MSVDSSSTHRHRSVAARLVRLAAAGAAVIAAVGTAAAWAHAGAVQHRCIHDAMQARVRQS
LdCL_100011000   1 MSVDSSSTHRHRSVAARLVRLAAAGAAVIAAVGTAAAWAHAGAVQHRCIHDAMQARVRQS
LdCL_100011100   1 MSVDSSSTHRHRSVAARLVRLAAAGAAVIAAVGTAAAWAHAGAVQHRCIHDAMQARVRQS
LINF_100010400   1 MSVDSSSTHRHRGVAARLVRLAAAGAAVIAAVGTAAAWAHAGAVQHRCIHDAMQARVRQS
LINF_100010200   1 MSVDSSSTHRHRSVAARLVRLAAAGAAVIAAVGTAAAWAHAGAVQHRCIHDAMQARVRQS
LINF_100011100   1 MSVDSSSTHRHRSVAARLVRLAAAGAAVIAAVGTAAAWAHAGAVQHRCIHDAMQARVRQS
LINF_100010300   1 MSVDSSSTHRHRSVAARLVRLAAAGAAVIAAVGTAAAWAHAGAVQHRCIHDAMQARVRQS
LINF_100010600   1 MSVDSSSTHRHRSVAARLVRLAAAGAAVIAAVGTAAAWAHAGAVQHRCIHDAMQARVRQS
LINF_100010800   1 MSVDSSSTHRHRSVAARLVRLAAAGAAVIAAVGTAAAWAHAGAVQHRCIHDAMQARVRQS
LINF_100010700   1 MSVDSSSTHRHRSVAARLVRLAAAGAAVIAAVGTAAAWAHAGAVQHRCIHDAMQARVRQS
LINF_100010900   1 MSVDSSSTHRHRSVAARLVRLAAAGAAVIAAVGTAAAWAHAGAVQHRCIHDAMQARVRQS
LINF_100011000   1 MSVDSSSTHRHRSVAARLVRLAAAGAAVIAAVGTAAAWAHAGAVQHRCIHDAMQARVRQS
```

```
LPMP_100440      58 VAQQRRPPGSVSALGLPYVSTGPISSAHTVDWALDDSTSPSVAHSADWDTMRIYVSYADL
LbrM.10.0480     58 VAQQHRPPGSVSALGLPYVSADPISSAHTVDWAQADSTSPSVAHSADWGTMRIYVSYADL
LbrM.10.0570      1 ------------------------------------------------------------
LPMP_100430      31 VAQQRRPPGSVSALGLPYVSTDPISSAHTVDWALADSTSPSVAHSADWGTLRILTSLEDL
LbrM.10.0470     51 VAQQRRPPGSVSALGLPYVSADPISSAHAVDWALADSTSPSVAHSPDWGTLRILTSLEDL
LbrM.10.0520     58 VAQQHRPPGSVSALGLPYVSA---------DWALADSTSPSVAHSADWGTLRILLSVEDL
LbrM.10.0540     40 VAQQHRPPGSVSALGLPYVSA---------DWALADSTSPSVAHSADWGTLRIFTSFEDL
LbrM.10.0590     61 AAHRRMPPSAVSAVGLX--------------XYIALDAADTVARAADWGTLRIAVSTADL
LPMP_100420      59 VAQQRRPPGSVSALGLPYVSTDPISSAHAVDWALADSTSPSVARAADWGTLRIAVSTADL
LbrM.10.0530      1 ------------------------------------------------------------
LbrM.10.0600     61 AAHRRMPPSAVSAV---------------GLPYIALDAADTVARAADWGTLRIAVSTADL
LPMP_100410      32 VAQQRRPPGSVSALGLPYVSTGPISSAHAVDWALADSTSPSVARAADWGTLRIAVSTADL
LbrM.10.0510     59 VAQQRRPPGSVSALGLPYVSADTISSAHTVDWAQADSTSPNVARAADWGTLRIAVSTADL
LbrM.10.0500      1 ------------------------------------------------------------
LbrM.10.0580     59 VAQQRSPAVSVSALGLPYVSAGTISSAHTVDWALADSTSPSVVRAADWGTLRIAVSTADL
LbrM.10.0610     58 VAQQRSPAVSVSALGLPYVSAGTISSAHTVDWALADSTSPSVVRAADWGTLRIAVSTADL
LbrM.10.0550     58 VAQQHMPPGSVSALGLPYVSAGTISSAHTVDWALADSTSPSVVRAADWGTLRIAVSTADL
LbrM.10.0560     59 VAQQRSPAVSVSALGLPYVSAGTISSAHTVDWALADSTSPSVVRAADWGTLRIAVSTADL
LtaP10.0480       9 VAHLGMAPSALSAVGLLFVTSDAAATSAAA-----EPGKFKVVRSRQWFTLRITVSTEDL
LtaP10.0650       1 -------------------------------------MAGSAVRAAEWGNLRIAVSTEDL
LmxM.10.0405     61 VAAQRMAPSAVSAVGLPHVTLDAADTAAGX-XDPSTGTPRNVVRAANWGALRIAVSAEDL
LmxM.10.0460     61 VAAQRMAPSAVSAVGLPYVSVVPVENASTLDYSLSDSTSPGVVRAANWGALRIAVSAEDL
LmxM.10.0465     61 VAAQRMAPSAVSAVGLPYVSVVPVENASTLDYSLSDSTSPGVVRAANWGALRIAVSAEDL
LmxM.10.0390     61 VAAQRMAPSAVSAVGLPHVTLDAADTAAGX-XDPSTGTPRNVVRAANWGALRIAVSAEDL
LmxM.10.0470     61 VAAQRMAPSAVSAVGLPHVTLDAADTAAGX-XDPSTGTPRNVVRAANWGALRIAVSAEDL
LINF_100011200   61 VARHHTAPGAVSAVGLPYVTLDTA---AAX-XDRRPGSAPTVVRAANWGALRIAVSTEDL
LdCL_100011300   61 VARHHTAPGAVSAVGLPYVTLDTA---AAX-XDRRPGSAPTVVRAANWGALRIAVSTEDL
LmjF.10.0480     61 VADHHKAPGAVSAVGLPYVTLDAAHTAAAX-XDPRPGSARSVVRDVNWGALRIAVSTEDL
LmjF.10.0460     61 VADHHKAPGAVSAVGLPYVTLDAAHTAAAX-XDPRPGSARSVVRDVNWGALRIAVSTEDL
LmjF.10.0465     61 VADHHKAPGAVSAVGLPYVTLDAAHTAAAX-XDPRPGSARSVVRDVNWGALRIAVSTEDL
LmjF.10.0470     61 VADHHKAPGAVSAVGLPYVTLDAAHTAAAX-XDPRPGSARSVVRDVNWGALRIAVSTEDL
LINF_100010100   61 VARHHTAPGAVSAVGLPYVTLDTA---AAX-XDRRPGSAPTVVRAANWGALRIAVSTEDL
LdCL_100010700   61 VARHHTAPGAVSAVGLPYVTLDTA---AAX-XDRRPGSAPTVVRAASWGALRIAVSTEDL
LdCL_100010500   61 VARHHTAPGAVSAVGLPYVTLDTA---AAX-XDRRPGSAPTVVRAANWGALRIAVSTEDL
LdCL_100010100   61 VARHHTAPGAVSAVGLPYVTLDTA---AAX-XDRRPGSAPTVVRAANWGALRIAVSTEDL
LdCL_100010300   61 VARHHTAPGAVSAVGLPYVTLDTA---AAX-XDRRPGSAPTVVRAANWGALRIAVSTEDL
LdCL_100011800   61 VARHHTAPGAVSAVGLSYVTLGAX--------------XPTVVRAANWGALRIAVSTEDL
LINF_100011300   61 VARHHTAPGAVSAVGLPYVTLDTA---AAX-XDRRPGSAPTVVRAANWGALRIAVSTEDL
LdCL_100011400   61 VARHHTAPGAVSAVGLPYVTLDTA---AAX-XDRRPGSAPTVVRAANWGALRIAVSTEDL
LdCL_100010900   61 VARHHTAPGAVSAVGLSYVTLGAX--------------XPTVVRAANWGALRIAVSTEDL
LdCL_100011600   61 VARHHTAPGAVSAVGLPYVTLDTA---AAX-XDRRPGSAPTVVRAASWGALRIAVSTEDL
LdCL_100011200   61 VARHHTAPGAVSAVGLPYVTLDTA---AAX-XDRRPGSAPTVVRAASWGALRIAVSTEDL
LdCL_100011500   61 VARHHTAPGAVSAVGLPYVTLDTA---AAX-XDRRPGSAPTVVRAASWGALRIAVSTEDL
LdCL_100011000   61 VARHHTAPGAVSAVGLPYVTLDTA---AAX-XDRRPGSAPTVVRAASWGALRIAVSTEDL
LdCL_100011100   61 VARHHTAPGAVSAVGLPYVTLDTA---AAX-XDRRPGSAPTVVRAASWGALRIAVSTEDL
LINF_100010400   61 VARHHTAPGAVSAVGLPYVTLDTA---AAX-XDRRPGSAPTVVRAANWGALRIAVSTEDL
LINF_100010200   61 VARHHTAPGAVSAVGLPYVTLDTA---AAX-XDRRPGSAPTVVRAANWGALRIAVSTEDL
LINF_100011100   61 VARHHTAPGAVSAVGLPYVTLDTA---AAX-XDRRPGSAPTVVRAANWGALRIAVSTEDL
LINF_100010300   61 VARHHTAPGAVSAVGLPYVTLDTA---AAX-XDRRPGSAPTVVRAANWGALRIAVSTEDL
LINF_100010600   61 VARHHTAPGAVSAVGLPYVTLDTA---AAX-XDRRPGSAPTVVRAANWGALRIAVSTEDL
LINF_100010800   61 VARHHTAPGAVSAVGLPYVTLDTA---AAX-XDRRPGSAPTVVRAANWGALRIAVSTEDL
LINF_100010700   61 VARHHTAPGAVSAVGLPYVTLDTA---AAX-XDRRPGSAPTVVRAANWGALRIAVSTEDL
LINF_100010900   61 VARHHTAPGAVSAVGLPYVTLDTA---AAX-XDRRPGSAPTVVRAANWGALRIAVSTEDL
LINF_100011000   61 VARHHTAPGAVSAVGLPYVTLDTA---AAX-XDRRPGSAPTVVRAANWGALRIAVSTEDL
LINF_100011000    1 MSVDSSSTHRHRSVAARLVRLAAAGAAVIAAVGTAAAWAHAGAVQHRCIHDAMQARVRQS
```

```
LPMP_100440      118 TDPDYYCSYVGQLIDNHDGAIDICEAEDILTDAKRDILVNVLLRQALQLHVERLKVRRVQ
LbrM.10.0480     118 IDPDYYCSYVGQLIDNHRGAIDICEAKDILTEEKRHILINILLPLALQLHVERLKVRQVQ
LbrM.10.0570       1 ------------------GAIDICEAKDILTEERRHILINILLPLALQLHVERLKVRQVQ
LPMP_100430       91 NDPDCYCFYVGQLVNNHQGAMDICDAEDILTEEKRNTLVNYLLPLALQLHVERLKVRQVQ
LbrM.10.0470     111 NDPDCYCSYVGQLIDNHQGAIDICEAEDVLTEEKRNILVTYLLPLALQLHVERLKVRQVQ
LbrM.10.0520     109 NDPDYYCSYVGQLVNNHAGALDMCKAEDILTDAKRNTLVGSLIPQALQLHTERLRVRQVQ
LbrM.10.0540      91 NDPDYYCSYVGQLVNNHAGALDMCKAEDILTDAKRNTLVGSIIPQALQLHTERLKVRQVQ
LbrM.10.0590     107 TDPDYHCTRVGQRVSNHADEIVTCTAEDVLTEEKRDILVSYLIPQALQLHAERLRVRQVQ
LPMP_100420      119 TDLCSHCTRVGQRVNNHNGEIATCTAEDILTEEKRDILVSYLIPQALQLHTERLKVKQVQ
LbrM.10.0530       1 -----DDASRRARVNNHADEIVTCTAEDILTEEKRDILVNYLIPQALQLHVERLRVRQVQ
LbrM.10.0600     106 TDPDYHCTRVGQRVSNHADEIVTCTAEDVLTEEKRDILVSYLIPQALQLHAERLRVRQVQ
LPMP_100410       92 NDPGYHCTRVGQRVNNHNGEIATCTAEDILTEQKRDILVSYLIPQALQLHAERLRVRQVQ
LbrM.10.0510     119 TDPGYHCTRVGQRVSNHADEIVTCTAEDVLTEEKRDILVSYLIPQALQLHAERLRVRQVQ
LbrM.10.0500       1 ------------------------------------------------------------
LbrM.10.0580     119 TDPDYYCSYVGQRVSNHAGAFVTCTAEDILTEEKRDILVTYLIPQALQLHVERLRVRQVQ
LbrM.10.0610     118 TDPDYYCSYVGQRVSNHAGAFVTCTAEDILTEEKRDILVTYLIPQALQLHVERLRVRQVQ
LbrM.10.0550     118 TDPDYYCTRVGQRVSNHAGAFVTCTAEDILTEEKRDILVTYLIPQALQLHVERLRVRQVQ
LbrM.10.0560     119 TDPDYHCTRVGQRVSNHAGAFVTCTAEDILTEEKRDILVSYLIPQALQLHVERLRVRQVQ
LtaP10.0480       64 TDPAYHCARVGQNISTHDGGLATCTADDILTDEKRDILVNYTLRQALQLHTDRLKVQQVQ
LtaP10.0650       24 TDPAYHCARVGQNVSTHDDGFATCTAKDILTDEKRDILVKDTIPQALQLHTDRLKVRQVQ
LmxM.10.0405     120 TDPAYHCARVGQRISARDGRFAVCTAEDILTDEKRDILVKHLVPQALQLHRERLKVRQVQ
LmxM.10.0460     121 TDPAYHCARVGQRVNNHAGDIVTCTAEDILTDEKRDTLVKHLVPQALQLHRERLKVRQVQ
LmxM.10.0465     121 TDPAYHCARVGQRVNNHAGDIVTCTAEDILTDEKRDTLVKHLVPQALQLHRERLKVRQVQ
LmxM.10.0390     120 TDPAYHCARVGQRVNNRVGDIVTCTAEDILTDEKRGILVKHLVPQALQLHRERLKVRQVQ
LmxM.10.0470     120 TDPAYHCARVGQRISARDGRFAVCTAEDILTDEKRDILVKHLVPQALQLHRERLKVRQVQ
LINF_100011200   117 TDPAYHCARVGQRVNNHAGAIATCTAEDILTDEKRDILVKYLIPQALQLHTERLKVRQVQ
LdCL_100011300   117 TDPAYHCARVGQRVNNHAGVIATCTAEDILTDEKRDILVKYLIPQALQLHTERLKVRQVQ
LmjF.10.0480     120 TDPAYHCARVGQHVKDHAGAIVTCTAEDILTNEKRDILVKHLIPQAVQLHTERLKVQQVQ
LmjF.10.0460     120 TDPAYHCARVGQHVKDHAGAIVTCTAEDILTNEKRDILVKHLIPQAVQLHTERLKVQQVQ
LmjF.10.0465     120 TDPAYHCARVGQHVKDHAGAIVTCTAEDILTNEKRDILVKHLIPQAVQLHTERLKVQQVQ
LmjF.10.0470     120 TDPAYHCARVGQRVNNHAGAIVTCTAEDILTNEKRNILVKYLIPQAVQLHKERLKVQQVQ
LINF_100010100   117 TDPAYHCARVGQHIKRRLGGVDICTAEDILTDEKRDILVKHLIPQALQLHTERLKVRQVQ
LdCL_100010700   117 TDPAYHCARVGQRVNTRTGAIATCTAEDILTDEKRDILVKYLIPQALQLHTERLKVRQVQ
LdCL_100010500   117 TDPAYHCARVGQRVNTRTGAIATCTAEDILTDEKRDILVKHLIPQALQLHTERLKVRQVQ
LdCL_100010100   117 TDPAYHCARVGQRVNTRTGAIATCTAEDILTDEKRDILVKYLIPQALELHTERLKVRQVQ
LdCL_100010300   117 TDPAYHCARVGQRVNTRTGAIATCTAEDILTDEKRDILVKYLIPQALQLHTERLKVRQVQ
LdCL_100011800   107 TDPAYHCARVGQRISTRDGRFAICTAKDILTDEKRDILVKHLIPQALQLHTERLKVRQVQ
LINF_100011300   117 TDPAYHCARVGQRISTHDSGSTTCTAEDILTDEKRDILVKHLIPQALQLHTERLKVRQVQ
LdCL_100011400   117 TDPAYHCARVGQRISTHDSGSTTCTAEDILTDEKRDILVKYLIPQALQLHTERLKVRQVQ
LdCL_100010900   107 TDPAYHCARVGQRISTRDGRFAICTAEDILTDEKRDILVKHLIPQALQLHTERLKVRQVQ
LdCL_100011600   117 TDPAYHCARVGQHIKRRLGGVDICTAEDILTDEKRDILVKHLIPQALQLHTERLKVRQVQ
LdCL_100011200   117 TDPAYHCARVGQHIKRRLGGVDICTAEDILTDEKRDILVKHLIPQALQLHTERLKVRQVQ
LdCL_100011500   117 TDPAYHCARVGQHIKRRLGGVDICTAEDILTDEKRDILVKHLIPQALQLHTERLKVRQVQ
LdCL_100011000   117 TDPAYHCARVGQHIKRRLGGVDICTAEDILTDEKRDILVKHLIPQALQLHTERLKVRQVQ
LdCL_100011100   117 TDPAYHCARVGQHIKRRLGGVDICTAEDILTDEKRDILVKHLIPQALQLHTERLKVRQVQ
LINF_100010400   117 TDPAYHCARVGQHIKRRLGGVDICTAEDILTDEKRDILVKHLIPQALQLHTERLKVRQVQ
LINF_100010200   117 TDPAYHCARVGQHIKRRLGGVDICTAEDILTDEKRDILVKHLIPQALQLHTERLKVRQVQ
LINF_100011100   117 TDPAYHCARVGQHIKRRLGGVDICTAEDILTDEKRDILVKHLIPQALQLHTERLKVRQVQ
LINF_100010300   117 TDPAYHCARVGQHIKRRLGGVDICTAEDILTDEKRDILVKHLIPQALQLHTERLKVRQVQ
LINF_100010600   117 TDPAYHCARVGQHIKRRLGGVDICTAEDILTDEKRDILVKHLIPQALQLHTERLKVRQVQ
LINF_100010800   117 TDPAYHCARVGQHIKRRLGGVDICTAEDILTDEKRDILVKHLIPQALQLHTERLKVRQVQ
LINF_100010700   117 TDPAYHCARVGQHIKRRLGGVDICTAEDILTDEKRDILVKHLIPQALQLHTERLKVRQVQ
LINF_100010900   117 TDPAYHCARVGQHIKRRLGGVDICTAEDILTDEKRDILVKHLIPQALQLHTERLKVRQVQ
LINF_100011000   117 TDPAYHCARVGQHIKRRLGGVDICTAEDILTDEKRDILVKHLIPQALQLHTERLKVRQVQ
LINF_100011000     1 MSVDSSSTHRHRSVAARLVRLAAAGAAVIAAVGTAAAWAHAGAVQHRCIHDAMQARVRQS
```

LPMP_100440	178	GSWKVTGMEGDVCGEFKVPEAHVTVGFSNTDFVLYVASVPSEGNILAWSAFCQVFTDGRP
LbrM.10.0480	178	GTWKVTGMEGDVCGEFKVPEEHVTVGFSNIDFVLYVASVPIEGNILAWSAFCQVFPDGRP
LbrM.10.0570	43	GTWKVTGMEGDVCGEFKVPEEHVTVGFSNIDFVLYVASVPSDTDVMAWSAFCQVFPDGRP
LPMP_100430	151	VTWKVTGMTGSICGEFKVPEAHVTIGVSNADFVLYVASVPSDPGVLAAAVICQTFSDSRP
LbrM.10.0470	171	GTWKVTGMEGDVCGTFKVPEEHVTVGVSNTDFVLYVASVPSEPGVLAAAVICQTFSDSRP
LbrM.10.0520	169	GSWKVTGMTGDVCGTFKVPKAHVTVGVSDADFVLYVASVPSEPGVLATAVICQTSSDGRP
LbrM.10.0540	151	GSWKVTGMTGDVCGTFKVPEAHVTVGVSDADFVLYVASVPSELGVLAWAVMCQAFFDDRP
LbrM.10.0590	167	GSWKVTGMTGDVCGEFKVPEAHVAKGVSNADFVLYVASVPSEPGVLAWATTCQVFSDDHP
LPMP_100420	179	GSWKVTGMTGSICGDYVVPTAHLTVGVSNADFVLYVASVPSEPGVLAWATICQVFSDKQP
LbrM.10.0530	56	GSWKVTGMMGRICGDYVVPTAHLTAGVSNADFVLYVASVPSEPGVLAWATICQMFSDGQP
LbrM.10.0600	166	GSWKVTGMTGDVCGEFKVPKAHVTVGVSNADFVLYVASVPSEPGVLAWATTCQVFSDDHP
LPMP_100410	152	GSWKVTGMTGSICGDFSVPPAHLTAGVSNADFVLYVASVPSEPGVLAWATTCQVFSDDHP
LbrM.10.0510	179	GSWKVTGMTGSICGDFSVPPAHLTAGVSNADFVLYVASVPSEPGVLAWATTCQVFSDDHP
LbrM.10.0500	1	---------------------MEGVSNADFVLYVASVPSEPGVLAWATTCQVFSDDHP
LbrM.10.0580	179	GSWRVTGMTGPICGDFKVPKAHVTVGVSNADFVLYVASVPSEPGVLAWATTCQVFSDDHP
LbrM.10.0610	178	GSWRVTGMTGPICGDFKVPKAHVTVGVSNADFVLYVASVPSEPGVLAWATTCQVFSDDHP
LbrM.10.0550	178	GSWRVTGMTGPICGDFKVPKAHVTVGVSNADFVLYVASVPSEPGVLAWATTCQMFSDDHP
LbrM.10.0560	179	GSWRVTGMTGPICGDFSVPTAHLTAGVSNADFVLYVASVPSEPGVLAWATTCQVFSDDHP
LtaP10.0480	124	GKWKVTGMVGEICGSFKVPEAHITEGLSNSDFVIYVTSVPSRPGVVAWAGTCQVFSDGYP
LtaP10.0650	84	GNWKVTGMVDDICGTFKVPEAHVTKGFRNTDFVLYVASVPSEPHVLAWATTCQLFSDGRP
LmxM.10.0405	180	GKWKVTDMAADVCSYFKVPPAHVTGGVSNTDFVLYVASVPSEESVLAWAMTCQVFPDGHP
LmxM.10.0460	181	GKWKVTGMAADVCGYFKVPPEHITEGVTNTDFVLYVASVPSEESVLAWATTCQVFADGHP
LmxM.10.0465	181	GKWKVTGMAADVCSYFKVPPEHITEGVTNTDFVLYVASVPSEESVLAWAMTCQVFPDGHP
LmxM.10.0390	180	GKWKVTGMAADVCSYFKVPPAHVTGGVSNTDFVLYVASVPSEESVLAWATTCQVFPDGHP
LmxM.10.0470	180	GKWKVTDMAADVCSYFKVPPAHVTGGVSNTDFVLYVASVPSEESVLAWAMTCQVFPDGHP
LINF_100011200	177	DKWNVTGMVDEICGDFKVPPAHITEGFSNTDFVMYVASVPSEEGVLAWATTCQVFSDGHP
LdCL_100011300	177	DKWKVTDMVDEICGDFKVPPAHITDGLSNTDFVMYVASVPSEEGVLAWATTCQVFSDGHP
LmjF.10.0480	180	GKWKVTDMVGEICGDFKVPQAHITEGFSNTDFVMYVASVPSEEGVLAWATTCQTFSDGHP
LmjF.10.0460	180	GKWKVTDMVGDICGDFKVPQAHITEGFSNTDFVMYVASVPSEEGVLAWATTCQTFSDGHP
LmjF.10.0465	180	GKWKVTDMVGDICGDFKVPQAHITEGFSNTDFVMYVASVPSEEGVLAWATTCQTFSDGHP
LmjF.10.0470	180	GKWKVTDMVGEICGDFKVPQAHITEGFSNTDFVMYVASVPSEEGVLAWATTCQTFSDGHP
LINF_100010100	177	DKWKVTGMGDDVCSDFKVPPAHITDGLSNTDFVMYVASVPSEGDVLAWAATCQVFSDGHP
LdCL_100010700	177	DKWKVTGMGDDVCSGFKVPQAHITDGLSNTDFVMYVASVPSEGDVLAWATTCQVFSDGHP
LdCL_100010500	177	DKWKVTGMGDDVCSDFKVPQAHITDGLSNTDFVMYVASVPSEGDVLAWATTCQVFSDGHP
LdCL_100010100	177	DKWKVTGMGDDVCSDFKVPQAHITDGLSNTDFVMYVASVPSEGDVLAWATTCQVFSDGHP
LdCL_100010300	177	DKWKVTGMGDDVCSDFKVPQAHITDGLSNTDFVMYVASVPSEGDVLAWATTCQVFSDGHP
LdCL_100011800	167	DKWKVTGMGDDVCSDFKVPPAHITDGLNNTDFVMYVASVPSEEDVLAWATTCQVFSDGHP
LINF_100011300	177	DKWKVTGMDDDVCSDFKVPPAHITDGLSNTDFVMYVASVPSEEGVLAWAATCQVFSDGHP
LdCL_100011400	177	DKWKVTGMVGDICGHFKVPPAHITDGLSNTDFVMYVASVPSEEGVLAWATTCQVFSDGHP
LdCL_100010900	167	DKWKVTGMGDDVCSDFKVPPAHITDGLSNTDFVMYVASVPSEEGVLAWATTCQVFSDGHP
LdCL_100011600	177	DKWKVTGMGDDVCSDFKVPPAHITDGLSNTDFVMYVASVPSEEGVLAWATTCQVFSDGHP
LdCL_100011200	177	DKWKVTGMGDDVCSDFKVPPAHITDGLSNTDFVMYVASVPSEEGVLAWATTCQVFSDGHP
LdCL_100011500	177	DKWKVTGMGDDVCSDFKVPPAHITDGLSNTDFVMYVASVPSEEGVLAWATTCQVFSDGHP
LdCL_100011000	177	DKWKVTGMGDDVCSDFKVPPAHITDGLSNTDFVMYVASVPSEEGVLAWATTCQVFSDGHP
LdCL_100011100	177	DKWKVTGMGDDVCSDFKVPPAHITDGLSNTDFVMYVASVPSEEGVLAWATTCQVFSDGHP
LINF_100010400	177	DKWKVTGMGDDVCSDFKVPPAHITDGLSNTDFVMYVASVPSEEGVLAWATTCQVFSDGHP
LINF_100010200	177	DKWKVTGMGDDVCSDFKVPPAHITDGLSNTDFVMYVASVPSEEGVLAWAATCQVFSDGHP
LINF_100011100	177	DKWKVTGMGDDVCSDFKVPPAHITDGLSNTDFVMYVASVPSEEGVLAWATTCQVFSDGHP
LINF_100010300	177	DKWKVTGMGDDVCSDFKVPPAHITDGLSNTDFVMYVASVPSEEGVLAWATTCQVFSDGHP
LINF_100010600	177	DKWKVTGMGDDVCSDFKVPPAHITDGLSNTDFVMYVASVPSEEGVLAWATTCQVFSDGHP
LINF_100010800	177	DKWKVTGMGDDVCSDFKVPPAHITDGLSNTDFVMYVASVPSEEGVLAWATTCQVFSDGHP
LINF_100010700	177	DKWKVTGMGDDVCSDFKVPPAHITDGLSNTDFVMYVASVPSEEGVLAWATTCQVFSDGHP
LINF_100010900	177	DKWKVTGMGDDVCSDFKVPPAHITDGLSNTDFVMYVASVPSEEGVLAWATTCQVFSDGHP
LINF_100011000	177	DKWKVTGMGDDVCSDFKVPPAHITDGLSNTDFVMYVASVPSEEGVLAWATTCQVFSDGHP
LINF_100011000	1	MSVDSSSTHRHRSVAARLVRLAAAGAAVIAAVGTAAAWAHAGAVQHRCIHDAMQARVRQS

```
LPMP_100440      238 AVGVINIPAANIRSAYDQIMVRTVAHEVAHALGFNRIFFDSAGMVAAAIGIRGKDYYAPV
LbrM.10.0480     238 AVGVINIPAAYIRSPYDQIMVRTVAHEVAHALGFNRIFFDSFGMVTAAIGIRGKDYYAPV
LbrM.10.0570     103 AVGVINIPAAHIRSPYDQLMIRTVAHEVAHALGFNRIFFDSFGMVTAAIGIRGKDYYAPV
LPMP_100430      211 AVGVINIPAANIRSAYDQIMVRTVAHEVAHALGFDLTLFDELELIHEVSDLRGKDYEVPV
LbrM.10.0470     231 AVGVINIPAANIRSPYDQLMVRTVTHEVAHTLGFDLTLFDELELIDEVNNLRGKDYEAPV
LbrM.10.0520     229 AVGVMNIPAAYIRSAYDQIMLRTVAHEVAHALGFDLTVFEDVGIVGDVSNLRGRDYEVPV
LbrM.10.0540     211 AVGVINIPAAYIQSAYDQFMLRSVTHEMAHALGFDLLLFEELDLLDVVSNLRGKDYEVPV
LbrM.10.0590     227 AVGVMNIPAANIVSRYDQGTTRTVTHEVAHALGFSSVFFEGTGIVKSVTNLRGKPFAAPV
LPMP_100420      239 AVAVINIPPANIHSPYDQVLTRAVAHEVAHALGFSRMYFERAGLLTNASHVRGKFFSVPV
LbrM.10.0530     116 AVGVMNIPAANIVSRDDQGLTRAVAHEVAHALGFSRMFFESTGIMMNVTNLRGRFFSVPV
LbrM.10.0600     226 AVGVMNIPAANIVSRYDQGTTRTVTHEVAHALGFSSVFFEGTGIVKSVTNLRGKPFAAPV
LPMP_100410      212 AVGVINIPAANIVSRYDQGATRVVTHEVAHALGFSSTFFKSAGIVKSVTNLRGKPFAAPV
LbrM.10.0510     239 AVGVMNIPAANIVSRYDQGTTRVVTHEVAHALGFSSVFFEGTGIVKSVTNLRGKPFAAPV
LbrM.10.0500      38 AVGVMNIPAANIVSRYDQGTTRTVTHEVAHALGFSSVFFEGTGIAKSVTNLRGKPFAAPV
LbrM.10.0580     239 AVGVMNIPAANIVSRYDQGTTRTVTHEVAHALGFSSVFFENAGIVTNVTNLRGKPFAAPV
LbrM.10.0610     238 AVGVMNIPAANIVSRYDQGTTRTVTHEVAHALGFSSVFFENAGIVMNVTNLRGKPFAAPV
LbrM.10.0550     238 AVGVMNIPAANIVSRYDQGTTRTVTHEVAHALGFSSVFFENAGIVKSVTNLRGKPFAAPV
LbrM.10.0560     239 AVGVMNIPAANIVSRYDQGTTRTVTHEVAHALGFSSVFFENAGIVTNVTNLRGKPFAAPV
LtaP10.0480      184 AVGVINIPAANIASRYNHAVTRAVAHEMAHVLGYSVHFMNAARIIKRKANVRGKPLSVPV
LtaP10.0650      144 AVGVINIPAASIASRYNQLVTRVVAHEMAHALGFSSTVFEAAGMLQSIPNVRGKPTLTPI
LmxM.10.0405     240 AVGVINIPAANIASRYDQLVTRVVAHEMAHALGFSGTFFEAVGIVQEVPGIRGKTFTVAV
LmxM.10.0460     241 AVGVINIPAANIASRYDQLVTRVVTHEMAHALGFSDTFFEAVGIVQEVPHVRGKDFNVSV
LmxM.10.0465     241 AVGVINIPAANIAARYDQLVTRVVTHEMAHALGFSDTFFEAVGIVQEVPHVRGKDFNVSV
LmxM.10.0390     240 AVGVINIPAANIAARYDQLVTRVVTHEMAHALGFSGTFFDAVGILRDVPNVRGKPYFALM
LmxM.10.0470     240 AVGVINIPAANIASRYDQLVTRVVAHEMAHALGFSGTFFEAVGIVQEVPGIRGKTFTVAV
LINF_100011200   237 AVGVINIPAANIASRYDQLVTRVVTHEMAHALGFSGTFFTEILLVTQMMNIRGKDFNVSV
LdCL_100011300   237 AVGVINIPAANIASRYDQLVTRVVTHEMAHALGFSGTFFTEILLVTQMMNIRGKDFNVSV
LmjF.10.0480     240 AVGVINIPAANIASRYDQLVTRVVTHEMAHALGFSGPFFEDARIVASVPNVRGKNFDVPV
LmjF.10.0460     240 AVGVINIPAANIASRYDQLVTRVVTHEMAHALGFSGPFFKDARIVASVPNVRGKNFDVPV
LmjF.10.0465     240 AVGVINIPAANIASRYDQLVTRVVTHEMAHALGFSGPFFEDARIVASVPNVRGKNFDVPV
LmjF.10.0470     240 AVGVINIPAANIASRYDQLVTRVVTHEMAHALGFSGPFFEDARIVANVSNVRGKNFDVPV
LINF_100010100   237 AVGVINIPAANIASRYDQLVTRVVTHEMAHALGFSDTFFTDKRMLHNVGKIRGKPHNAPV
LdCL_100010700   237 AVGVINIPAANIASRYNQLVTRVVTHEMAHALGFSDTFFTDKRMLHNLEKIRGKPHNAPV
LdCL_100010500   237 AVGVINIPAANIASRYNQLVTRVVTHEMAHALGFSDTFFTDKRMLHNLEKIRGKPHNAPV
LdCL_100010100   237 AVGVINIPAANIASRYNQLVTRVVTHEMAHALGFSDTFFTDKRMLHNLEKIRGKPHNAPV
LdCL_100010300   237 AVGVINIPAANIASRYNQLVTRVVTHEMAHALGFSDTFFTDKRMLHNLEKIRGKPHNAPV
LdCL_100011800   227 AVGVINIPAANIASRYDQLVTRVVTHEMAHTLGFSVVFFRDASIMHQVSNIRRKTSKVPV
LINF_100011300   237 AVGVVNIPAANIASRYNQLVTRVVTHEMAHTLGFSVDFFQDASIMHQVSNIRRKTSKVPV
LdCL_100011400   237 AVGVINIPAANIASRYDQLVTRVVTHEMAHALGFSVGFFEGARILENISNVRHKDFDVPV
LdCL_100010900   227 AVGVINIPAANIASRYDQLVTRVVTHEMAHALGFSVGFFEGARILENISNVRHKDFDVPV
LdCL_100011600   237 AVGVINIPAANIASRYDQLVTRVVTHEMAHALGFSVGFFEGARILENISNVRHKDFDVPV
LdCL_100011200   237 AVGVINIPAANIASRYDQLVTRVVTHEMAHALGFSVGFFEGARILENISNVRHKDFDVPV
LdCL_100011500   237 AVGVINIPAANIASRYDQLVTRVVTHEMAHALGFSVGFFEGARILENISNVRHKDFDVPV
LdCL_100011000   237 AVGVINIPAANIASRYDQLVTRVVTHEMAHALGFSVGFFEGARILENISNVRHKDFDVPV
LdCL_100011100   237 AVGVINIPAANIASRYDQLVTRVVTHEMAHALGFSVGFFEGARILENISNVRHKDFDVPV
LINF_100010400   237 AVGVINIPAANIASRYDQLVTRVVTHEMAHALGFSVGFFEGARILESISNVRHKDFDVPV
LINF_100010200   237 AVGVINIPAANIASRYDQLVTRVVTHEMAHALGFSVGFFEGARILESISNVRHKDFDVPV
LINF_100011100   237 AVGVINIPAANIASRYDQLVTRVVTHEMAHALGFSVGFFEGARILESISNVRHKDFDVPV
LINF_100010300   237 AVGVINIPAANIASRYDQLVTRVVTHEMAHALGFSVGFFEGARILESISNVRHKDFDVPV
LINF_100010600   237 AVGVINIPAANIASRYDQLVTRVVTHEMAHALGFSVGFFEGARILESISNVRHKDFDVPV
LINF_100010800   237 AVGVINIPAANIASRYDQLVTRVVTHEMAHALGFSVGFFEGARILESISNVRHKDFDVPV
LINF_100010700   237 AVGVINIPAANIASRYDQLVTRVVTHEMAHALGFSVGFFEGARILESISNVRHKDFDVPV
LINF_100010900   237 AVGVINIPAANIASRYDQLVTRVVTHEMAHALGFSVGFFEGARILESISNVRHKDFDVPV
LINF_100011000   237 AVGVINIPAANIASRYDQLVTRVVTHEMAHALGFSVGFFEGARILESISNVRHKDFDVPV
LINF_100011000     1 MSVDSSSTHRHRSVAARLVRLAAAGAAVIAAVGTAAAWAHAGAVQHRCIHDAMQARVRQS
```

```
LPMP_100440      298 INSSTVVAKAREQYGCTPLSFLELEDTGSPASLGSHLKGRNAKDELMAPAVTAGYYTALT
LbrM.10.0480     298 LNTPTVVTKAREQYGCTLLSFLELEDTGGPASLGSHLKGRNAKDELMSSVVKGGYYTALT
LbrM.10.0570     163 LNTPTVVTKAREQYGCTLLSFLELEDTGGPASLGSHLKGRNAKDELMSSVVKGGYYTALT
LPMP_100430      271 LNSPTVMAKAREQYGCPTLEYLELEDTGGGSAAGSHLKGRNAQDELMAPVSAAGYYTALT
LbrM.10.0470     291 LSSPTVVAKAREQYGCPTLEFLELEDTGGGSAAGSHLKGRNAKDELMAPVSAAGYYTALT
LbrM.10.0520     289 LSSPTVAAKAREQYGCATLTYLELEDTGGGSTAGSHLKMRNAKEELMAPAVTAGYYTALT
LbrM.10.0540     271 LNSPTVVAKAREQYGCATLTFLEVEDTGDGSTAGSHLKRRNAKDELMAPVMGAGYYTALT
LbrM.10.0590     287 INSSTAVAKAREQYGCPTLEYLEVEDQGGSGSAGSHLKGRNAKDELMAPASAAGYYTNLT
LPMP_100420      299 MNSSTVVAKAREQYGCSNLTYVELEDLGDSRTTASHLKGRNAKDELMAPASAAGYYTALT
LbrM.10.0530     176 LNSSTAVAKAREQYGCTNLTYLELEDTGGCSTAGSHLKGRNAKDELMAPASAAGYYTNLT
LbrM.10.0600     286 INSSTAVAKAREQYGCPTLEYLEVEDQGGSGSAGSHLKGRNAKDELMAPASAAGYYTNLT
LPMP_100410      272 INSSTVVAKAREQYGCPTLEYLEVEDQGGSGSAGSHIKMRNAKDELMAPASAAGYYTALT
LbrM.10.0510     299 INSSTAVAKAREQYGCPTLEYLEVEDQGGSGSAGSHLKGRNAKDELMAPASAAGYYTNLT
LbrM.10.0500      98 INSSTVVAKAREQYGCPTLEYLEVEDQGGSGSAGSHLKGRNAKDELMAPASAAGYYTNLT
LbrM.10.0580     299 INSSTVVAKAREQYGCATLEYLEVEDQGGSGSAGSHLKGRNAKDELMAPASAAGYYTALT
LbrM.10.0610     298 INSSTVVAKAREQYGCPTLEYLEVEDQGGSGSAGSHLKGRNAKDELMAPASAAGYYTALT
LbrM.10.0550     298 INSSTAVAKAREQYGCPTLEYLEVEDQGGSGSAGSHLKGRNAKDELMAPASAAGYYTALT
LbrM.10.0560     299 INSSTAVAKAREQYGCPTLEYLEVEDQGGSGSAGSHLKGRNAKDELMAPASAAGYYTALT
LtaP10.0480      244 IEANRVVVRAREQYGCDNLKYLELEDQGGEGSVQSHIKMRNAQDELMAADASVGYYTAMT
LtaP10.0650      204 MNSSTVVAKAREQYGCETLEHLELEDHGGSGSAGSHIKMRNAKDELMAPAAGGGYYTALT
LmxM.10.0405     300 ITSSTAVAKAREQYGCNSLEYLEIEDQGGAGSAGSHIKMRNAKDELMAPAASAGYYTALT
LmxM.10.0460     301 ITSSTAVAKAREQYGCNSLEYLEIEDQGGAGSAGSHIKMRNAQDELMAPAASAGYYTALT
LmxM.10.0465     301 ITSSTVVAKAREQYGCCCLWYLEIEDQGGAGSAGSHIKMRNAQDELMAPAASAGYYTALT
LmxM.10.0390     300 INSSTAVAKAREQYGCNSLEYLEMEDQGDAGSAGSHIKMRNAQDELMAASASAGYYTALT
LmxM.10.0470     300 INSSTAVAKAREQYGCNSLEYLEMEDQGGAGSAGSHIKMRNAQDELMAPAASAGYYTALT
LINF_100011200   297 INSSTAVAKAREQYGCDTLEYLEIEDQGGAGSAGSHIKMRNAKDELMAPAAAAGYYSALT
LdCL_100011300   297 INSSTAVAKAREQYGCGTLEYLEIEDQGGAGSAGSHIKMRNAKDELMAPAAAAGYYSALT
LmjF.10.0480     300 INSSTAVAKAREQYGCDTLEYLEMEDQGSAGSAGSHIKMRNAQDELMAAASGAGYYTALT
LmjF.10.0460     300 INSSTAVAKAREQYGCDTLEYLEVEDQGGAGSAGSHIKMRNAQDELMAPAAAAGYYTALT
LmjF.10.0465     300 INSSTAVAKAREQYGCDTLEYLEVEDQGGAGSAGSHIKMRNAQDELMAPAAAAGYYTALT
LmjF.10.0470     300 INSSTAVAKAREQYGCDTLEYLEVEDQGGAGSAGSHIKMRNAQDELMAPAAAAGYYTALT
LINF_100010100   297 INSSTAVAKAREQYGCDTLEYLEMEDEGGAVSAGSHIKMRNAQDELMAPAAAAGYYSALT
LdCL_100010700   297 IHSSTAVAKAREQYGCGTLEYLEMEDEGGAVSAGSHIKMRNAQDELMAPAAAAGYYSALT
LdCL_100010500   297 IHSSTAVAKAREQYGCGTLEYLEMEDEGGAVSAGSHIKMRNAQDELMAPAAAAGYYSALT
LdCL_100010100   297 IHSSTAVAKAREQYGCGTLEYLEMEDEGGAVSAGSHIKMRNAQDELMAPAAAAGYYSALT
LdCL_100010300   297 IHSSTAVAKAREQYGCGTLEYLEMEDEGGAVSAGSHIKMRNAQDELMAPAAAAGYYSALT
LdCL_100011800   287 LKSRTAVAKAREQYGCGTLEYLEMEDQGGAGSAGSHIKMRNAQDELMAPASDAGYYSALT
LINF_100011300   297 LKSRTAVAKAREQYGCDTLEYLEIEDQGGAGSAGSHIKMRNAQDELMAPAAAAGYYSALT
LdCL_100011400   297 INSSTAVAKAREQYGCDTLEYLEIEDQGGAGSAGSHIKMRNAQDELMAPAAAAGYYSALT
LdCL_100010900   287 INSSTAVAKAREQYGCGTLEYLEIEDQGGAGSAGSHIKMRNAQDELMAPAAAAGYYSALT
LdCL_100011600   297 INSSTAVAKAREQYGCDTLEYLEIEDQGGAGSAGSHIKMRNAQDELMAPASDAGYYSALT
LdCL_100011200   297 INSSTAVAKAREQYGCDTLEYLEIEDQGGAGSAGSHIKMRNAQDELMAPAAAAGYYSALT
LdCL_100011500   297 INSSTAVAKAREQYGCDTLEYLEIEDQGGAGSAGSHIKMRNAQDELMAPAAAAGYYSALT
LdCL_100011000   297 INSSTAVAKAREQYGCDTLEYLEIEDQGGAGSAGSHIKMRNAQDELMAPAAAAGYYSALT
LdCL_100011100   297 INSSTAVAKAREQYGCDTLEYLEIEDQGGAGSAGSHIKMRNAQDELMAPAAAAGYYSALT
LINF_100010400   297 INSSTAVAKAREQYGCDTLEYLEIEDQGGAGSAGSHIKMRNAQDELMAPAAAAGYYSALT
LINF_100010200   297 INSSTAVAKAREQYGCDTLEYLEIEDQGGAGSAGSHIKMRNAQDELMAPAAAAGYYSALT
LINF_100011100   297 INSSTAVAKAREQYGCDTLEYLEIEDQGGAGSAGSHIKMRNAQDELMAPAAAAGYYSALT
LINF_100010300   297 INSSTAVAKAREQYGCDTLEYLEIEDQGGAGSAGSHIKMRNAQDELMAPAAAAGYYSALT
LINF_100010600   297 INSSTAVAKAREQYGCDTLEYLEIEDQGGAGSAGSHIKMRNAQDELMAPAAAAGYYSALT
LINF_100010800   297 INSSTAVAKAREQYGCDTLEYLEIEDQGGAGSAGSHIKMRNAQDELMAPAAAAGYYSALT
LINF_100010700   297 INSSTAVAKAREQYGCDTLEYLEIEDQGGAGSAGSHIKMRNAQDELMAPAAAAGYYSALT
LINF_100010900   297 INSSTAVAKAREQYGCDTLEYLEIEDQGGAGSAGSHIKMRNAQDELMAPAAAAGYYSALT
LINF_100011000   297 INSSTAVAKAREQYGCDTLEYLEIEDQGGAGSAGSHIKMRNAQDELMAPAAAAGYYSALT
LINF_100011000     1 MSVDSSSTHRHRSVAARLVRLAAAGAAVIAAVGTAAAWAHAGAVQHRCIHDAMQARVRQS
```

LPMP_100440	358	LAVFEDFGFYQADFAKAEVMPWANLATCDFLTKKCMEKNITQWPGMFCNSTDXXLLSCST
LbrM.10.0480	358	MAVFQDLGFYQADFSMAEVMPWAYLATCDFLTNKCMERNITQWPGMFCNTTDXXLLRCST
LbrM.10.0570	223	MAVFQDLGFYQADFSMAEVMPWAYLASCDFLTNKCMERNITQWPGMFCNTTDXXLLRCST
LPMP_100430	331	MAVFEDLGFYKADFAKAEMMPWANLATCDFLTKKCMENNITQWPWMFCNTTEXXSYRCPT
LbrM.10.0470	351	MAVFQDLGFYRADFTKAEVMPWANLASCDFLTNKCMERNITQWPGMFCNTTEXXSYRCTS
LbrM.10.0520	349	MAVFQDLGFYQVDFTKAEVMPWAYLASCDFITKKCMEDNITQWPGMFCNSTEXXLYRCTS
LbrM.10.0540	331	MATFEDFGFYRVDFTKAEVMPWAEHASCDFITNKCMEDNITQWPEMFCNSTEXXSYRCPT
LbrM.10.0590	347	MAVFEDLGFYKADFTKAEVMPWGRNASCDFLTKKCMERNITQWPEMFCNTDEXXALRCPT
LPMP_100420	359	MAVFEDLGFYKADYNLAEIMPWAQNASCDFLTKKCMENNITQWPEMFCNTTEXXVPQCPT
LbrM.10.0530	236	MAVFEDLGFYKADFSMAEVMPWGRNASCDFLTNKCMEDNITQWPEMFCNTTKXXVSQCPT
LbrM.10.0600	346	MAVFEDLGFYKADFTKAEVMPWGRNASCDFLTNKCMEDNITQWPEMFCNTTEXXRYRCPT
LPMP_100410	332	MAVFEDLGFYKADFAKAEVMPWGRNASCDFLTQKCMEDNITQWPEMFCNTTEXXRYRCPT
LbrM.10.0510	359	MAVFEDLGFYKADFSMAEVMPWGRNASCDFLTNKCMEDNITQWPEMFCNTTEXXRYRCPT
LbrM.10.0500	158	MAVFEDLGFYKADFTKAEVMPWGRNASCDFLTKKCMEDNITQWPEMFCNTTDENALRCTT
LbrM.10.0580	359	MAVFEDLGFYKADFSMAEVMPWGRNASCDFITNKCMEDNITQWPEMFCNTTEXXRYRCPT
LbrM.10.0610	358	MAVFEDLGFYKADFSMAEVMPWGRNASCDFITNKCMEDNITQWPEMFCNTTEXXRYRCPT
LbrM.10.0550	358	MAVFEDLGFYKADFSMAEVMPWGRTASCDFITNKCMEDNITQWPEMFCNTTDENALRCTT
LbrM.10.0560	359	MAVFEDLGFYKADFTKAEVMPWGRNASCNFLTKKCMEDNITQWPEMFCNTTDENALRCTT
LtaP10.0480	304	MAIFEALRFYRADFRRAEVMPWGQNTGCAFLSEKCMENNITKWPKMFCNSTEXXMFRCPT
LtaP10.0650	264	MAIFQDLGFYQADFSKAEEMPWGKDAGCAFLSEKCMEKNITKWPSMFCDGKRXXEARCPT
LmxM.10.0405	360	MAVFQDLGFYQADFSKAEEMPWGRNVRCAFLSEKCMAKNVTKWPAMFCNESAXXTIRCPT
LmxM.10.0460	361	MAVFQDLGFYQADFSKAEAMPWGRNAGCAFLSEKCMAKNVTKWPPMFCNESAXXTIRCPT
LmxM.10.0465	361	MAVFQDLGFYQADFSKAEAMPWGRNAGCAFLSEKCMAKNVTKWPAMFCNESAXXAIRCPT
LmxM.10.0390	360	MAVFQDLGFYQADFSKAEAMPWGRNAGCAFLSEKCMENGITKWPAMFCNESAXXAIRCPT
LmxM.10.0470	360	MAVFQDLGFYQADFSKAEAMPWGRNAGCAFLSEKCMENGVTKWPAMFCNESAXXAIRCPT
LINF_100011200	357	MAIFQDLGFYQADFSKAEVMPWGRNAGCAFLSEKCMERNITKWPAMFCNENEXXTMRCPT
LdCL_100011300	357	MAIFQDLGFYQADFSKAEEMPWGRNAGCAFLSEKCMEQNITKWPAMFCNENEXXTMRCPT
LmjF.10.0480	360	MAILQDLGFYQADFSKAEVMPWGQNAGCAFLTNKCMEQNITQWPAMFCNESEXXAIRCPT
LmjF.10.0460	360	MAIFQDLGFYQADFSKAEVMPWGQNAGCAFLTNKCMEQSVTQWPAMFCNESEXXAIRCPT
LmjF.10.0465	360	MAIFQDLGFYQADFSKAEVMPWGQNAGCAFLTNKCMEQSVTQWPAMFCNESEXXAIRCPT
LmjF.10.0470	360	MAIFQDLGFYQADFSKAEVMPWGQNAGCAFLTNKCMEQNITQWPAMFCNESEXXAIRCPT
LINF_100010100	357	MAIFQDLGFYQADFSKAEVMPWGRNAGCAFLSEKCMEDGITKWPAMFCNSDX-XALRCPT
LdCL_100010700	357	MAIFQDLGFYQADFSKAEEMPWGRNAGCAFLSEKCMEQNITKWPAMFCNDAX-XSIGCPT
LdCL_100010500	357	MAIFQDLGFYQADFSKAEEMPWGRNAGCAFLSEKCMEQNITKWPAMFCNDAX-XSIGCPT
LdCL_100010100	357	MAIFQDLGFYQADFSKAEEMPWGRNAGCAFLSEKCMEQNITKWPAMFCNDAX-XSIGCPT
LdCL_100010300	357	MAIFQDLGFYQADFSKAEEMPWGRNAGCAFLSEKCMEQNITKWPAMFCNDAX-XSIGCPT
LdCL_100011800	347	MAIFQDLGFYQADFSKAEEMPWGRNAGCAFLSEKCMEQNITKWPAMFCNIDX-XAVRCPT
LINF_100011300	357	MAIFQDLGFYQADFSKAEVMPWGRNAGCAFLSEKCMERNITKWPAMFCNENEXXTMRCPT
LdCL_100011400	357	MAIFQDLGFYQADFSKAEEMPWGRNAGCAFLSEKCMEDGITKWPAMFCNENEXXTMRCPT
LdCL_100010900	347	MAIFQDLGFYQADFSKAEEMPWGRNAGCAFLSEKCMEDGITKWPAMFCNENEXXTMRCPT
LdCL_100011600	357	MAIFQDLGFYQADFSKAEEMPWGRNAGCAFLSEKCMEDGITKWPAMFCNENEXXTMRCPT
LdCL_100011200	357	MAIFQDLGFYQADFSKAEEMPWGRNAGCAFLSEKCMEDGITKWPAMFCNENEXXTMRCPT
LdCL_100011500	357	MAIFQDLGFYQADFSKAEEMPWGRNAGCAFLSEKCMEDGITKWPAMFCNENEXXTMRCPT
LdCL_100011000	357	MAIFQDLGFYQADFSKAEVMPWGRNAGCAFLSEKCMEDGITKWPAMFCNENEXXTMRCPT
LdCL_100011100	357	MAIFQDLGFYQADFSKAEVMPWGRNAGCAFLSEKCMEDGITKWPAMFCNENEXXTMRCPT
LINF_100010400	357	MAIFQDLGFYQADFSKAEVMPWXRNAGCAFLSEKCMERNITKWPAMFCNENEXXX-----
LINF_100010200	357	MAIFQDLGFYQADFSKAEVMPWGRNAGCAFLSEKCMERNITKWPAMFCNENEXXTMRCPT
LINF_100011100	357	MAIFQDLGFYQADFSKAEVMPWGRNAGCAFLSEKCMERNITKWPAMFCNENEXXTMRCPT
LINF_100010300	357	MAIFQDLGFYQADFSKAEVMPWGRNAGCAFLSEKCMERNITKWPAMFCNENEXXTMRCPT
LINF_100010600	357	MAIFQDLGFYQADFSKAEVMPWGRNAGCAFLSEKCMERNITKWPAMFCNENEXXTMRCPT
LINF_100010800	357	MAIFQDLGFYQADFSKAEVMPWGRNAGCAFLSEKCMERNITKWPAMFCNENEXXTMRCPT
LINF_100010700	357	MAIFQDLGFYQADFSKAEVMPWGRNAGCAFLSEKCMERNITKWPAMFCNENEXXTMRCPT
LINF_100010900	357	MAIFQDLGFYQADFSKAEVMPWGRNAGCAFLSEKCMERNITKWPAMFCNENEXXTMRCPT
LINF_100011000	357	MAIFQDLGFYQADFSKAEVMPWGRNAGCAFLSEKCMERNITKWPAMFCNENEXXTMRCPT
LINF_100011000	1	MSVDSSSTHRHRSVAARLVRLAAAGAAVIAAVGTAAAWAHAGAVQHRCIHDAMQARVRQS

```
LPMP_100440      418 DRLTLGTCKFTQRKRPLPLYFQYFTEPSLGGFSSFMDFCPFVDTYPNGACNQDPSMASPT
LbrM.10.0480     418 DRLTLGTCKFTQRKRPLPTYFQYFTDSFIGGFSPFMDYCPYVDTYPDGACNQDPSMASPS
LbrM.10.0570     283 DRLTLGTCKFTQRKRPLPTYFQYFTDSFIGGFSPFMDYCPYVDTYPDGACNQDPSMASPS
LPMP_100430      391 HRLKIGRCSIVVYDDPLPTYLRYFTEPSVGGRLPFMDLCPIIVGYGTAACNQDPSTASPT
LbrM.10.0470     411 DRLKIGRCSITTYDDPMPTYFRYFTETSVGGRISFMDYCPVIVGYGTAACNQDPSTASPT
LbrM.10.0520     409 DRLKIGRCIIVTHDDPLPTYFQYFTESSVGGRLTFMDFCPLVMGYSNTACDQDPSTAPSF
LbrM.10.0540     391 QRLKIGRCSIATYDDPLPTYFQYFTKTSLGGSSEFMDFCPIIVGYSNIACNQDPSTASPA
LbrM.10.0590     407 DRLGLGGCIVLTHXXSVPQYFQYFTDPTLTGLSDFMDYCPTVVPYDDGSCAQRASETSSD
LPMP_100420      419 DRLRLGTCLIIDVGRPMSMIYQYFTNDSLSGRSPFLDYCPVIVGSSNSACDQDPSMASPS
LbrM.10.0530     296 DRLRLGTCLIISDGRPMAPYYQYFNDTSLGGLSPFLDHCPVIVASSDGACNQNPSMASPF
LbrM.10.0600     406 DRLRLGTCGIRTYSTPMPTYFQYFNDTFLAGYSAFLDYCPFTLGYRNGACNQDPSTAPAL
LPMP_100410      392 DRLRLGTCGIRTYSTPMPPYFEYFNDTFLAGYSAFLDYCPFTLGYSNGACNQDPSTAPAL
LbrM.10.0510     419 DRLRLGTCGIRTYSTPVPTYFQYFNDTFLAGYSAFLDYCPFTLGYRNGACNQDPSTAPAL
LbrM.10.0500     218 DRLRLGTCGIRTYSTPMPTYFQYFTNAFLGGFSEFLDYCPFIVGYSNGACNQDPSTASPA
LbrM.10.0580     419 DRLKLGTCGIRTYSTPMPTYFQYFTNAFLGGFSAFLDYCPFIVDYSNGACNQDPSMASPS
LbrM.10.0610     418 DRLKLGTCGIRTYSTPMPTYFQYFTNAFLGGFSEFLDYCPFIVDYSNGACNQDPSMASPS
LbrM.10.0550     418 DRLKLGTCGIRTYSTPMPTYFQYFTNASLGGFSAFLDFCPFIVGYSNGACNQDPSTASPS
LbrM.10.0560     419 DRLKLGTCGIRTYSTPMPTYFQYFTNAFLGGFSAFLDYCPFIVGYSNGACNQDPSTASPA
LtaP10.0480      364 NRLGIGSCVMRPQEAPLPNHFAYFTIPLLSGSSPLRDYCPVVEDEGEGSCAQNASQAPAA
LtaP10.0650      324 SRISLGSCGVTTXXEELPAHFQYFTDPLLSGNVPFMDYCPAVMLSNNGDCAQSASGASPI
LmxM.10.0405     420 DRLRVGTCGITAYNTSLATYWQYFTNASLGGYSPFLDYCPFVVGYRNGSCNQDASTTPDL
LmxM.10.0460     421 DRLSLGTCGITAYNTSLATYWQYFTNASLGGYSPFLDYCPTVVGYRNGSCNQDASTAPDL
LmxM.10.0465     421 DRLSLGTCGITAYNTSLATYWQYFTNASLGGDSAFLDYCPTVVDYSDGSCNQDASTAPDL
LmxM.10.0390     420 SRLSVGMCDVTRXXQALPPYLQYFTDPFLAGSSAFMDYCPAVVPFADGNCGQSASEADAA
LmxM.10.0470     420 SRLSVGMCDVTPXXQALPPYLQYFTDPFLAGSSAFMDYCPVVVPYDDGSCGQSASEADAA
LINF_100011200   417 SRLMVGTCGIRGYSTPFSLYWQYFTNASLGGYSPFLDYCPFVIGYSDGSCNQDASLAAGF
LdCL_100011300   417 GRLMLGTCGIRGYSTPFSPYWQYFTNISLGGYSPFLDYCPFVIGYGDGSCNQDASLATGF
LmjF.10.0480     420 SRLSLGACGVTR-HPGLPPYWQYFTDPSLAGVSAFMDYCPVVVPYSDISCTQRASEAHAS
LmjF.10.0460     420 SRLSLGACGVTR-HPGLPPYWQYFTDPSLAGLSAFMDYCPVVVPYSDGSCTQRASEAHAS
LmjF.10.0465     420 SRLSLGACGVTR-HPGLPPYWQYFTDPSLAGLSAFMDYCPVVVPYSDGSCTQRASEAHAS
LmjF.10.0470     420 SRLLLGTCGIREYELPLPRYWQYFTNASLGGYSPFLDYCPFVIDYADGSCNQDASSAEEF
LINF_100010100   416 SRLSLGACSLAT-YQSLPPYWQYFTDPSLAGISAFMDYCPVVVPFGNGSCAQNASKVMAA
LdCL_100010700   416 GRLSLGACSLGT-YQSLPQYWQYFTDPSLAGISAFMDYCPVVVPFGDGSCAQNASKVIAA
LdCL_100010500   416 GRLSLGACSLGT-YQSLPQYWQYFTDPSLAGISAFMDYCPVVVPFGDGSCAQNASKVIAA
LdCL_100010100   416 GRLSLGACSLGT-YQSLPQYWQYFTDPSLAGISAFMDYCPVVVPFGDGSCAQNASKVIAA
LdCL_100010300   416 GRLSLGACSLGT-YQSLPQYWQYFTDPSLAGISAFMDYCPVVVPFGDGSCAQNASKVIAA
LdCL_100011800   406 SRLSLGVCGIAFQRFPMPPYWQYFTDPLLAGISAFMDYCPVVVPYGDGSCAQNTSEADLA
LINF_100011300   417 SRLSLGKCGVTR-HPDLPPYWQYFTDPSLAGISAFMDCCPVVEPYGDGSCAQRASEAGAP
LdCL_100011400   417 SRLGLGKCGVTR-HPDLPPYWQYFTDPSLAGISAFMDYCPVVEPYGDGSCAQRASEAGAP
LdCL_100010900   407 SRLGLGKCGVTR-HPDLPPYWQYFTDPSLAGISAFMDCCPVVEPYGDGSCAQRASEAGAP
LdCL_100011600   417 SRLGLGKCGVTR-HPDLPPYWQYFTDPSLAGISAFMDYCPVVEPYGDGSCAQRASEAGAP
LdCL_100011200   417 SRLGLGKCGVTR-HPDLPPYWQYFTDPSLAGISAFMDYCPVVEPYGDGSCAQRASEAGAP
LdCL_100011500   417 SRLGLGKCGVTR-HPDLPPYWQYFTDPSLAGISAFMDYCPVVEPYGDGSCAQRASEAGAP
LdCL_100011000   417 SRLGLGKCGVTR-HPDLPPYWQYFTDPSLAGISAFMDCCPVVEPYGDGSCAQRASEAGAP
LdCL_100011100   417 SRLGLGKCGVTR-HPDLPPYWQYFTDPSLAGISAFMDYCPVVEPYGDGSCAQRASEAGAP
LINF_100010400       ------------------------------------------------------------
LINF_100010200   417 SRLSLGKCGVTR-HPDLPPYWQYFTDPSLAGISAFMDCCPVVEPYGDGSCAQRASEAGAP
LINF_100011100   417 SRLSLGKCGVTR-HPDLPPYWQYFTDPSLAGISAFMDCCPVVEPYGDGSCAQRASEAGAP
LINF_100010300   417 SRLSLGKCGVTR-HPDLPPYWQYFTDPSLAGISAFMDCCPVVEPYGDGSCAQRASEAGAP
LINF_100010600   417 SRLSLGKCGVTR-HPDLPPYWQYFTDPSLAGISAFMDCCPVVEPYGDGSCAQRASEAGAP
LINF_100010800   417 SRLSLGKCGVTR-HPDLPPYWQYFTDPSLAGISAFMDCCPVVEPYGDGSCAQRASEAGAP
LINF_100010700   417 SRLSLGKCGVTR-HPDLPPYWQYFTDPSLAGISAFMDCCPVVEPYGDGSCAQRASEAGAP
LINF_100010900   417 SRLSLGKCGVTR-HPDLPPYWQYFTDPSLAGISAFMDCCPVVEPYGDGSCAQRASEAGAP
LINF_100011000   417 SRLSLGKCGVTR-HPDLPPYWQYFTDPSLAGISAFMDCCPVVEPYGDGSCAQRASEAGAP
LINF_100011000     1 MSVDSSSTHRHRSVAARLVRLAAAGAAVIAAVGTAAAWAHAGAVQHRCIHDAMQARVRQS
```

```
LPMP_100440      478 VKEFNLFSDAARCFDGVYRPMHGIVHGNHYNGLCAKVKCDRAHHMYSVRVYGSSDYVACT
LbrM.10.0480     478 LMEFNVFSDAARCLDGVFRPKHGIVHGNHYNGLCANVKCDRVHHRYSVQVYGSSGYVACT
LbrM.10.0570     343 LMEFNVFSDAARCLDGVFRPKHGIVHGNHYNGLCANVKCDRVHHRYSVQVYGSSGYVACT
LPMP_100430      451 VKKFSLFSDSSRCFDGVFTPKHSTGPPGPYNGLCANVKCDRX------------------
LbrM.10.0470     471 VKEFSLFSDSSRCLDGNFAPKHNTGPSDHYNSLCANVKCDRAHHTYSVQVYGSSGYVACT
LbrM.10.0520     469 FKEFSLFSDSSRCFDGTFRPKHSTGPPGPYNGMCAKVKCDRAHHTYSVQVRGSSGYVACT
LbrM.10.0540     451 LMEFNVFSDSSRCFDGTLTPKHNTGPYEQYNALCANVMCDRAHHTYSVEVRGSSGYVACT
LbrM.10.0590     467 MQAFNVFSDAARCLDGAFRPTTTXXEDVTYAGMCANVKCDTAARTYSVQVRGSSGYVACT
LPMP_100420      479 LQAFNLFSDAARCFDGVFQLRNNTPSQEPYNALCANVKCDRAHHMYSVQVYGSRGYVACT
LbrM.10.0530     356 LQAFNVFSDAARCFDGVFQPRNSTTPSPKYNALCANVKCDRDHHTYSVQVRGSSGYVACT
LbrM.10.0600     466 FKEFSVFSDAARCLDGVFQPRNSTTPSPKYNGMCAKVKCDRAHHTYSVQVRGSSGYVACT
LPMP_100410      452 LKEFSVFSDAARCFDGAFRPTTARXXDLMYNALCANVMCNTAARTYSVQVRGSSGYVACT
LbrM.10.0510     479 FKEFSVFSDAARCLDGVFQPRNSTTPSPKYNALCANVKCDRDHHTYSVEVRGSSGYVACT
LbrM.10.0500     278 LMEFNVFSDAARCLDGAFRPTTTRXXYVTYAGMCANVMCDTAARTYSVQVRGSSDYVACT
LbrM.10.0580     479 LQAFNVFSDAARCFDGVFQPRNSNARSEPNNALCANVMCDTAARTYSVQVRGSSGYVACT
LbrM.10.0610     478 LQAFNVFSDAARCFDGVFQPRNSNARSEPNNALCANVMCDTAARTYSVQVRGSSGYVACT
LbrM.10.0550     478 LKEFNVFSDAARCLDGVFQPRNSNARSEPNNALCANVMCDTAARTYSVQVRGSSGYVACT
LbrM.10.0560     479 LMEFNVFSDAARCLDGVFQPRNSNARSEPNNALCANVMCDTAARTYSVQVRGSSGYVACT
LtaP10.0480      424 MKAFNVFSDAARCIDGDFRPKGMQSPAKSHAGLCANVRCDPATRTYSVQVRGSSGYMNCV
LtaP10.0650      384 LNIFNVFSDAARCIDGAFSFQNPQGSRSSASAMCANVRCDTATRTYSVQVRGSKRYVDCT
LmxM.10.0405     480 LAAFNVFSEAARCIDGAFTPKNRTAADGYYTALCANVKCDTATRTYSVQVRGSNGYANCT
LmxM.10.0460     481 LAAFNVFSETARCIDGAFTPKNRTAADGYYGGLCANVKCDTATRTYSVQVRGSNGYVNCT
LmxM.10.0465     481 FAAFNVFSETARCIDGAFTPKNRTAADGYYGGLCANVKCDTVTRTYSVQVRGSNGYVNCT
LmxM.10.0390     480 FKAFNVFSDAARCIDGAFRPKTTHGLIKSYAALCANVKCDTATRTYSVQVRGSSGYANCT
LmxM.10.0470     480 FKAFNVFSDAARCIDGAFRPKTTHGVIKSYAALCANVKCDTAARTYSVQVRGSSGYANCT
LINF_100011200   477 FSAFNVFSDAARCIDGAFRPKNRTAANGYYAGLCANVRCDTATRTYSVQVRGSMDYVNCT
LdCL_100011300   477 FGAFNVFSDAARCIDGAFRPKNRTAADGYYAGLCANVRCDTATRTYSVQVCGSMDYVNCT
LmjF.10.0480     479 LLPFNVFSDAARCIDGAFRPKATNGIVKSYAGLCANVQCDTATRTYSVQVHGSNDYTNCT
LmjF.10.0460     479 LLPFNVFSDAARCIDGAFRPKATDGIVKSYAGLCANVQCDTATRTYSVQVHGSNDYTNCT
LmjF.10.0465     479 LLPFNVFSDAARCIDGAFRPKATNGIVKSYAGLCANVQCDTATRTYSVQVHGSNDYTNCT
LmjF.10.0470     480 FTAFNVFSDAARCIDGAFRPKATNGIVKSYAGLCANVQCDTATRTYSVQVHGSNDYTNCT
LINF_100010100   475 VQAFNVFSDAARCIDGAFRPKTTETVTNSYAGLCANVRCDTATRTYSVQVRGGSGYASCT
LdCL_100010700   475 VQAFNVFSDAARCIDGAFRPKTSHGIIKSYAGLCANVRCDTATRTYSVQVHGGSGYANCT
LdCL_100010500   475 VQAFNVFSDAARCIDGAFRPKTSHGIIKSYAGLCANVRCDTATRTYSVQVHGGSGYANCT
LdCL_100010100   475 VQAFNVFSDAARCIDGAFRPKTSHGIIKSYAGLCANVRCDTATRTYSVQVHGGSGYANCT
LdCL_100010300   475 VQAFNVFSDAARCIDGAFRPKTSHGIIKSYAGLCANVRCDTATRTYSVQVHGGSGYANCT
LdCL_100011800   466 LKSFSVFSDAARCIDGAFRPKTSHGIIKSYAGLCANVRCDTATRTYSVQVHGGSGYANCT
LINF_100011300   476 FKGFNVFSDAARCIDGAFRPKTSHGIIKSYAGLCANVRCDTATRTYSVQVHGGSGYANCT
LdCL_100011400   476 FKGFNVFSDAARCIDGAFRPKTSHGIIKSYAGLCANVRCDTATRTYSVQVHGGSGYANCT
LdCL_100010900   466 FKGFNVFSDAARCIDGAFRPKTSHGIIKSYAGLCANVRCDTATRTYSVQVHGGSGYANCT
LdCL_100011600   476 FKGFNVFSDAARCIDGAFRPKTSHGIIKSYAGLCANVRCDTATRTYSVQVHGGSGYANCT
LdCL_100011200   476 FKGFNVFSDAARCIDGAFRPKTSHGIIKSYAGLCANVRCDTATRTYSVQVHGGSGYANCT
LdCL_100011500   476 FKGFNVFSDAARCIDGAFRPKTSHGIIKSYAGLCANVRCDTATRTYSVQVHGGSGYANCT
LdCL_100011000   476 FKGFNVFSDAARCIDGAFRPKTSHGIIKSYAGLCANVRCDTATRTYSVQVHGGSGYANCT
LdCL_100011100   476 FKGFNVFSDAARCIDGAFRPKTSHGIIKSYAGLCANVRCDTATRTYSVQVHGGSGYANCT
LINF_100010400       ------------------------------------------------------------
LINF_100010200   476 FKGFNVFSDAARCIDGAFRPKTSHGQIKSYAGLCANVRCDTATRTYSVQVHGGSGYANCT
LINF_100011100   476 FKGFNVFSDAARCIDGAFRPKTSHGQIKSYAGLCANVRCDTATRTYSVQVHGGSGYANCT
LINF_100010300   476 FKGFNVFSDAARCIDGAFRPKTSHGIIKSYAGLCANVRCDTATRTYSVQVHGGSGYANCT
LINF_100010600   476 FKGFNVFSDAARCIDGAFRPKTSHGIIKSYAGLCANVRCDTATRTYSVQVHGGSGYANCT
LINF_100010800   476 FKGFNVFSDAARCIDGAFRPKTSHGIIKSYAGLCANVRCDTATRTYSVQVHGGSGYANCT
LINF_100010700   476 FKGFNVFSDAARCIDGAFRPKTSHGIIKSYAGLCANVRCDTATRTYSVQVHGGSGYANCT
LINF_100010900   476 FKGFNVFSDAARCIDGAFRPKTSHGIIKSYAGLCANVRCDTATRTYSVQVHGGSGYANCT
LINF_100011000   476 FKGFNVFSDAARCIDGAFRPKTSHGIIKSYAGLCANVRCDTATRTYSVQVHGGSGYANCT
LINF_100011000     1 MSVDSSSTHRHRSVAARLVRLAAAGAAVIAAVGTAAAWAHAGAVQHRCIHDAMQARVRQS
```

```
LPMP_100440      538 PGERVELATTSAAFVEGSYIVCASYVEVCQANIKGVIDFEGDAADTAAVX----------
LbrM.10.0480     538 PGQSIELATTSDAFVEGSYIMCPLYVEVCQANIKGVIDFEGDAADTAAVX----------
LbrM.10.0570     403 PGQSIELATTSTAFVEGSYIMCPLYVEVCQANIKGVIDFEGDAADTAAAX----------
LPMP_100430          ------------------------------------------------------------
LbrM.10.0470     531 PGESVELATISTAFVEGSYIICASYVEVCQANIKGLIDFEGDAADTAAVX----------
LbrM.10.0520     529 PGERLDLATLSTTFVEGSYIMCPPYVEVCQANIKGVIDFERDAADTAAVX----------
LbrM.10.0540     511 PGERVELTTISTAFVEGSYITCAPYVEVCQANIKGVIDFERDAADTAAVX----------
LbrM.10.0590     527 PGESVELATLSAAFVNGSYITCAPYVEVCQANVQGATSSGNAAAGRRGPRX--------X
LPMP_100420      539 PGESVELATLSAAFENGGNITCPPYVEVCQANTKGLIDFEGDAADTAAMRX--------X
LbrM.10.0530     416 PGERVDLATLSAAFVNGSYITCAPYVEVCQANIKGVIDFEGDAADTAAMRX--------X
LbrM.10.0600     526 PGERLDLATLSTAFVEGSYIMCPPYVEVCQANIKGVIDFEGDAADTAAMRX--------X
LPMP_100410      512 PGESVELATTSAAFVEGSYITCAPYVEVCQANIKGVIDFEGDAADTAAMRX--------X
LbrM.10.0510     539 PGESVELATISTAFVNGSYITCAPYVEVCQANIKGLIDFERDAADTAAMRX--------X
LbrM.10.0500     338 PGESVDLATLSAAFVEGTYITCPLYVEVCQANIKGVIDFERDAADTAAVX----------
LbrM.10.0580     539 PGESIDLATLSAAFVNGSYITCAPYVEVCQANIKGLIDFERDAADTAAMRX--------X
LbrM.10.0610     538 PGESIDLATLSAAFVNGSYITCAPYVEVCQANIKGLIDFERDAADTAAMRX--------X
LbrM.10.0550     538 PGESIDLATLSAAFVEGSYIMCPPYVEVCQANIKGVIDFERDAADTAAMRX--------X
LbrM.10.0560     539 PGESIDLATLSAAFVEGSYITCPPYVEVCQANIKGVIDFERDAADTAAMRX--------X
LtaP10.0480      484 PGLRVELSSVSDAFEQGGYITCPPYVEVCQGNAGAVKGVGNGVPSX--------------
LtaP10.0650      444 PGLRVELSSVSDAFEQGGYITCPPYVEVCQGNLQGAKESNNAVACYRGPRAASAALLVAA
LmxM.10.0405     540 PGLRVKLSSVSDAFEKGGYVTCPPYVEVCQGNVKAAKDFAGDTDSSSSADDAADKEAMQR
LmxM.10.0460     541 PGLRVKLSSVSDAFEKGGYVTCPPYVEVCQGNVKAAKDFAGDTDSSSSADDAADKEAMQR
LmxM.10.0465     541 PGLRVKLSSVSDAFEKGGYVTCPPYVEVCQGNVKAAKDFAGDTDSSSSADDAADKEAMQR
LmxM.10.0390     540 PGLRFELSTVSDAFEKGGYVTCPPYVEVCQGNPQAIKDGGNAAAGRRGPRAAATALVVAA
LmxM.10.0470     540 PGLRFELSTVSDAFEKGGYVTCPPYVEVCQGNAQAIKDGGNAAAGRRGPRAAATALVVAA
LINF_100011200   537 PGLRVELSTVSSAFEEGGYITCPPYVEVCQANVKGAKDFAGDSDSSSSAGDAADRAAMQR
LdCL_100011300   537 PGLRVELSTVSSAFEEGGYITCPPYVEVCQANVKGAKDFAGDSDSSSSAGDAADRAAMQR
LmjF.10.0480     539 PGLRVELSTVSNAFEGGGYITCPPYVEVCQGNVQAAKDGGNTAAGRRGPRAAATALLVAA
LmjF.10.0460     539 PGLRVELSTVSNAFEGGGYITCPPYVEVCQGNVQAAKDGGNTAAGRRGPRAAATALLVAA
LmjF.10.0465     539 PGLRVELSTVSNAFEGGGYITCPPYVEVCQGNVQAAKDGGNTAAGRRGPRAAATALLVAA
LmjF.10.0470     540 PGLRVELSTVSKTFEEGGYITCPPYVEVCQGNVQAAKDFDGDSDSSSSSSDAADKAAIER
LINF_100010100   535 PGLRVELSTVSSAFEEGGYITCPPYVEVCQGNVQAAKDGGNAAAGRRGPRAAATALLXGR
LdCL_100010700   535 PGLRVELSTVSSAFEEGGYITCPPYVEVCQGNVQAAKDGGNAAAGRRGPRAAATALLXGR
LdCL_100010500   535 PGLRVELSTVSSAFEEGGYITCPPYVEVCQGNVQAAKDGGNAAAGRRGPRAAATALLXGR
LdCL_100010100   535 PGLRVELSTVSSAFEEGGYITCPPYVEVCQGNVQAAKDGGNAAAGRRGPRAAATALLXGR
LdCL_100010300   535 PGLRVELSTVSSAFEEGGYITCPPYVEVCQGNVQAAKDGGNAAAGRRGPRAAATALLXGR
LdCL_100011800   526 PGLRVELSTVSSAFEEGGYITCPPYVEVCQGNVQAAKDGGNAAAGRRGPRAAATALLXGR
LINF_100011300   536 PGLRVELSTVSSAFEEGGYITCPPYVEVCQGNVQAAKDGGNAAAGRRGPRAAATALLXGR
LdCL_100011400   536 PGLRVELSTVSSAFEEGGYITCPPYVEVCQGNVQAAKDGGNAAAGRRGPRAAATALLXGR
LdCL_100010900   526 PGLRVELSTVSSAFEEGGYITCPPYVEVCQGNVQAAKDGGNAAAGRRGPRAAATALLXGR
LdCL_100011600   536 PGLRVELSTVSSAFEEGGYITCPPYVEVCQGNVQAAKDGGNAAAGRRGPRAAATALLXGR
LdCL_100011200   536 PGLRVELSTVSSAFEEGGYITCPPYVEVCQGNVQAAKDGGNAAAGRRGPRAAATALLXGR
LdCL_100011500   536 PGLRVELSTVSSAFEEGGYITCPPYVEVCQGNVQAAKDGGNAAAGRRGPRAAATALLXGR
LdCL_100011000   536 PGLRVELSTVSSAFEEGGYITCPPYVEVCQGNVQAAKDGGNAAAGRRGPRAAATALLXGR
LdCL_100011100   536 PGLRVELSTVSSAFEEGGYITCPPYVEVCQGNVQAAKDGGNAAAGRRGPRAAATALLXGR
LINF_100010400       ------------------------------------------------------------
LINF_100010200   536 PGLRVELSTVSSAFEEGGYITCPPYVEVCQGNVQAAKDGGNAAAGRRGPRAAATALLXGR
LINF_100011100   536 PGLRVELSTVSSAFEEGGYITCPPYVEVCQGNVQAAKDGGNAAAGRRGPRAAATALLXGR
LINF_100010300   536 PGLRVELSTVSSAFEEGGYITCPPYVEVCQGNVQAAKDGGNAAAGRRGPRAAATALLXGR
LINF_100010600   536 PGLRVELSTVSSAFEEGGYITCPPYVEVCQGNVQAAKDGGNAAAGRRGPRAAATALLXGR
LINF_100010800   536 PGLRVELSTVSSAFEEGGYITCPPYVEVCQGNVQAAKDGGNAAAGRRGPRAAATALLXGR
LINF_100010700   536 PGLRVELSTVSSAFEEGGYITCPPYVEVCQGNVQAAKDGGNAAAGRRGPRAAATALLXGR
LINF_100010900   536 PGLRVELSTVSSAFEEGGYITCPPYVEVCQGNVQAAKDGGNAAAGRRGPRAAATALLXGR
LINF_100011000   536 PGLRVELSTVSSAFEEGGYITCPPYVEVCQGNVQAAKDGGNAAAGRRGPRAAATALLXGR
LINF_100011000     1 MSVDSSSTHRHRSVAARLVRLAAAGAAVIAAVGTAAAWAHAGAVQHRCIHDAMQARVRQS
```

```
LPMP_100440             ------------------------------------------------------------
LbrM.10.0480            ------------------------------------------------------------
LbrM.10.0570            ------------------------------------------------------------
LPMP_100430             ------------------------------------------------------------
LbrM.10.0470            ------------------------------------------------------------
LbrM.10.0520            ------------------------------------------------------------
LbrM.10.0540            ------------------------------------------------------------
LbrM.10.0590       579  XRX----TALLVAALLAIACAX-------------------------------------X
LPMP_100420        591  W----------------------------------------------------------X
LbrM.10.0530       468  WRERMTALATVTAALLGIVLAAMAGLAVWLLLISLPX----------------------X
LbrM.10.0600       578  WRERMTALATVTAALLGIVLAAMAGLAVWLLLITIPX----------------------X
LPMP_100410        564  WSERMTALATVTAVLLGIVLAVMAILVVWLLLITIPX----------------------X
LbrM.10.0510       591  WRERMTALATVTAALLGIVLAAMAGLVVGLLVISLSX----------------------X
LbrM.10.0500            ------------------------------------------------------------
LbrM.10.0580       591  WSERMYVLATVTAVLLGIVLAAMAGLVVGLLVISLPX----------------------X
LbrM.10.0610       590  WSERMYVLATVTAVLLGIVLAAMAGLVVGLLVISLSX----------------------X
LbrM.10.0550       590  WSERMYVLATVTAVLLGIVLAAMAGLVSV------------------------------X
LbrM.10.0560       591  WSERMTALATVTAVLLGIVLAAMAGLVVGLLVISLSX----------------------X
LtaP10.0480             ------------------------------------------------------------
LtaP10.0650        504  LLSITCMX-------------------------------------------------VAA
LmxM.10.0405       600  WSDRMAALATATTLLLGMVLSLMALLVVRLLLTSSPWCCCRLGGLPTX---------MQR
LmxM.10.0460       601  WSDRVAALATATTLLLGMVLSLVTLLVVRLLLTSSPWCCCRLGGGAPDVSCDGPLAXMQR
LmxM.10.0465       601  WSDRVAALATATTLLLGMVLSLVTLLVVRLLLTSSPWCCCRLGGLPTX---------MQR
LmxM.10.0390       600  XXARRGALX------------------------------------------------VAA
LmxM.10.0470       600  XXARRGALX------------------------------------------------VAA
LINF_100011200     597  WNDRMAGLATAAMVLLGMVLSLMALVVVWLLLLTCPWWCCKFGGLPTX---------MQR
LdCL_100011300     597  WNDRMAGLATAAMVLLGMVLSLMALVVVWLLLLTCPWWCCKFGGLPTX---------MQR
LmjF.10.0480       599  -XAGRGALX------------------------------------------------VAA
LmjF.10.0460       599  -XADRGALX------------------------------------------------VAA
LmjF.10.0465       599  -XADRGALX------------------------------------------------VAA
LmjF.10.0470       600  WNERMAGLATAATVLLGVVLSLMALVVVWLLLVSCPRWCCKVGGLPTX---------IER
LINF_100010100     595  XXAGRGALX------------------------------------------------XGR
LdCL_100010700     595  XXAGRGALX------------------------------------------------XGR
LdCL_100010500     595  XXAGRGALX------------------------------------------------XGR
LdCL_100010100     595  XXAGRGALX------------------------------------------------XGR
LdCL_100010300     595  XXAGRGALX------------------------------------------------XGR
LdCL_100011800     586  XXAGRGALX------------------------------------------------XGR
LINF_100011300     596  XXAGRGALX------------------------------------------------XGR
LdCL_100011400     596  XXAGRGALX------------------------------------------------XGR
LdCL_100010900     586  XXAGRGALX------------------------------------------------XGR
LdCL_100011600     596  XXAGRGALX------------------------------------------------XGR
LdCL_100011200     596  XXAGRGALX------------------------------------------------XGR
LdCL_100011500     596  XXAGRGALX------------------------------------------------XGR
LdCL_100011000     596  XXAGRGALX------------------------------------------------XGR
LdCL_100011100     596  XXAGRGALX------------------------------------------------XGR
LINF_100010400          ------------------------------------------------------------
LINF_100010200     596  XXAGRGALX------------------------------------------------XGR
LINF_100011100     596  XXAGRGALX------------------------------------------------XGR
LINF_100010300     596  XXAGRGALX------------------------------------------------XGR
LINF_100010600     596  XXAGRGALX------------------------------------------------XGR
LINF_100010800     596  XXAGRGALX------------------------------------------------XGR
LINF_100010700     596  XXAGRGALX------------------------------------------------XGR
LINF_100010900     596  XXAGRGALX------------------------------------------------XGR
LINF_100011000     596  XXAGRGALX------------------------------------------------XGR
LINF_100011000       1  MSVDSSSTHRHRSVAARLVRLAAAGAAVIAAVGTAAAWAHAGAVQHRCIHDAMQARVRQS
```

```
LPMP_100440              ------------------------------------------------------------
LbrM.10.0480             ------------------------------------------------------------
LbrM.10.0570             ------------------------------------------------------------
LPMP_100430              ------------------------------------------------------------
LbrM.10.0470             ------------------------------------------------------------
LbrM.10.0520             ------------------------------------------------------------
LbrM.10.0540             ------------------------------------------------------------
LbrM.10.0590         579 XRX----TALLVAALLAIACAX-------------------------------------X
LPMP_100420          591 W----------------------------------------------------------X
LbrM.10.0530         468 WRERMTALATVTAALLGIVLAAMAGLAVWLLLISLPX----------------------X
LbrM.10.0600         578 WRERMTALATVTAALLGIVLAAMAGLAVWLLLITIPX----------------------X
LPMP_100410          564 WSERMTALATVTAVLLGIVLAVMAILVVWLLLITIPX----------------------X
LbrM.10.0510         591 WRERMTALATVTAALLGIVLAAMAGLVVGLLVISLSX----------------------X
LbrM.10.0500             ------------------------------------------------------------
LbrM.10.0580         591 WSERMYVLATVTAVLLGIVLAAMAGLVVGLLVISLPX----------------------X
LbrM.10.0610         590 WSERMYVLATVTAVLLGIVLAAMAGLVVGLLVISLSX----------------------X
LbrM.10.0550         590 WSERMYVLATVTAVLLGIVLAAMAGLVSV------------------------------X
LbrM.10.0560         591 WSERMTALATVTAVLLGIVLAAMAGLVVGLLVISLSX----------------------X
LtaP10.0480              ------------------------------------------------------------
LtaP10.0650          504 LLSITCMX-------------------------------------------------VAA
LmxM.10.0405         600 WSDRMAALATATTLLLGMVLSLMALLVVRLLLTSSPWCCCRLGGLPTX---------MQR
LmxM.10.0460         601 WSDRVAALATATTLLLGMVLSLVTLLVVRLLLTSSPWCCCRLGGGAPDVSCDGPLAXMQR
LmxM.10.0465         601 WSDRVAALATATTLLLGMVLSLVTLLVVRLLLTSSPWCCCRLGGLPTX---------MQR
LmxM.10.0390         600 XXARRGALX------------------------------------------------VAA
LmxM.10.0470         600 XXARRGALX------------------------------------------------VAA
LINF_100011200       597 WNDRMAGLATAAMVLLGMVLSLMALVVVWLLLLTCPWWCCKFGGLPTX---------MQR
LdCL_100011300       597 WNDRMAGLATAAMVLLGMVLSLMALVVVWLLLLTCPWWCCKFGGLPTX---------MQR
LmjF.10.0480         599 -XAGRGALX------------------------------------------------VAA
LmjF.10.0460         599 -XAGRGALX------------------------------------------------VAA
LmjF.10.0465         599 -XADRGALX------------------------------------------------VAA
LmjF.10.0470         600 WNERMAGLATAATVLLGVVLSLMALVVVWLLLVSCPRWCCKVGGLPTX---------IER
LINF_100010100       595 XXAGRGALX------------------------------------------------XGR
LdCL_100010700       595 XXAGRGALX------------------------------------------------XGR
LdCL_100010500       595 XXAGRGALX------------------------------------------------XGR
LdCL_100010100       595 XXAGRGALX------------------------------------------------XGR
LdCL_100010300       595 XXAGRGALX------------------------------------------------XGR
LdCL_100011800       586 XXAGRGALX------------------------------------------------XGR
LINF_100011300       596 XXAGRGALX------------------------------------------------XGR
LdCL_100011400       596 XXAGRGALX------------------------------------------------XGR
LdCL_100010900       586 XXAGRGALX------------------------------------------------XGR
LdCL_100011600       596 XXAGRGALX------------------------------------------------XGR
LdCL_100011200       596 XXAGRGALX------------------------------------------------XGR
LdCL_100011500       596 XXAGRGALX------------------------------------------------XGR
LdCL_100011000       596 XXAGRGALX------------------------------------------------XGR
LdCL_100011100       596 XXAGRGALX------------------------------------------------XGR
LINF_100010400           ------------------------------------------------------------
LINF_100010200       596 XXAGRGALX------------------------------------------------XGR
LINF_100011100       596 XXAGRGALX------------------------------------------------XGR
LINF_100010300       596 XXAGRGALX------------------------------------------------XGR
LINF_100010600       596 XXAGRGALX------------------------------------------------XGR
LINF_100010800       596 XXAGRGALX------------------------------------------------XGR
LINF_100010700       596 XXAGRGALX------------------------------------------------XGR
LINF_100010900       596 XXAGRGALX------------------------------------------------XGR
LINF_100011000       596 XXAGRGALX------------------------------------------------XGR
LINF_100011000         1 MSVDSSSTHRHRSVAARLVRLAAAGAAVIAAVGTAAAWAHAGAVQHRCIHDAMQARVRQS
```
