# Supplementary material for: Variation in Leishmania chemokine suppression driven by diversification of the GP63 virulence factor
Source: PLoS Negl Trop Dis. 2021 Oct 28;15(10):e0009224. doi: 10.1371/journal.pntd.0009224 (PMC8577781; doi:10.1371/journal.pntd.0009224)
Supplement: S2 Fig — Residues adjacent to the CXCL10 binding site demonstrate a pattern of exclusive mutation between the Leishmania and Viannia subgenera. The EBF generated by MEME was plotted onto the phylogenetic tree for positively selected residues (at a threshold of p<0.1) within 5 amino acids of the predicted CXCL10 binding site. The phylogenetic tree was rooted at the node of the most recent common ancestor of the two Sauroleishmania sequences identified. The amino acid residue for each sequence at the indicated position is plotted based on a multisequence alignment generated in ClustalOmega and used for both evolutionary tests above. MEME excludes identical sequences; therefore only 49 of 54 identified sequences are displayed in the phylogeny. P-values calculated based on likelihood ratio test statistic (LRT) as described in Murrell et al. 2012 [45]. Tip labels include species and genomic location. For species labels: “LBRM” is L. (V.) braziliensis MHOM/BR/75/M2904, “LDCL” is L. (L.) donovani CL-SL, “LINF” is L. (L.) infantum JPCM5, “LMJF” is L. (L.) major strain Friedlin, “LMXM” is L. (L.) mexicana MHOM/GT/2001/U1103, “LPMP” is L. (V.) panamensis strain MHOM/PA/94/PSC-1, and “LTAP” is L. tarentolae Parrot-TarII. (DOCX) [file pntd.0009224.s004.docx]

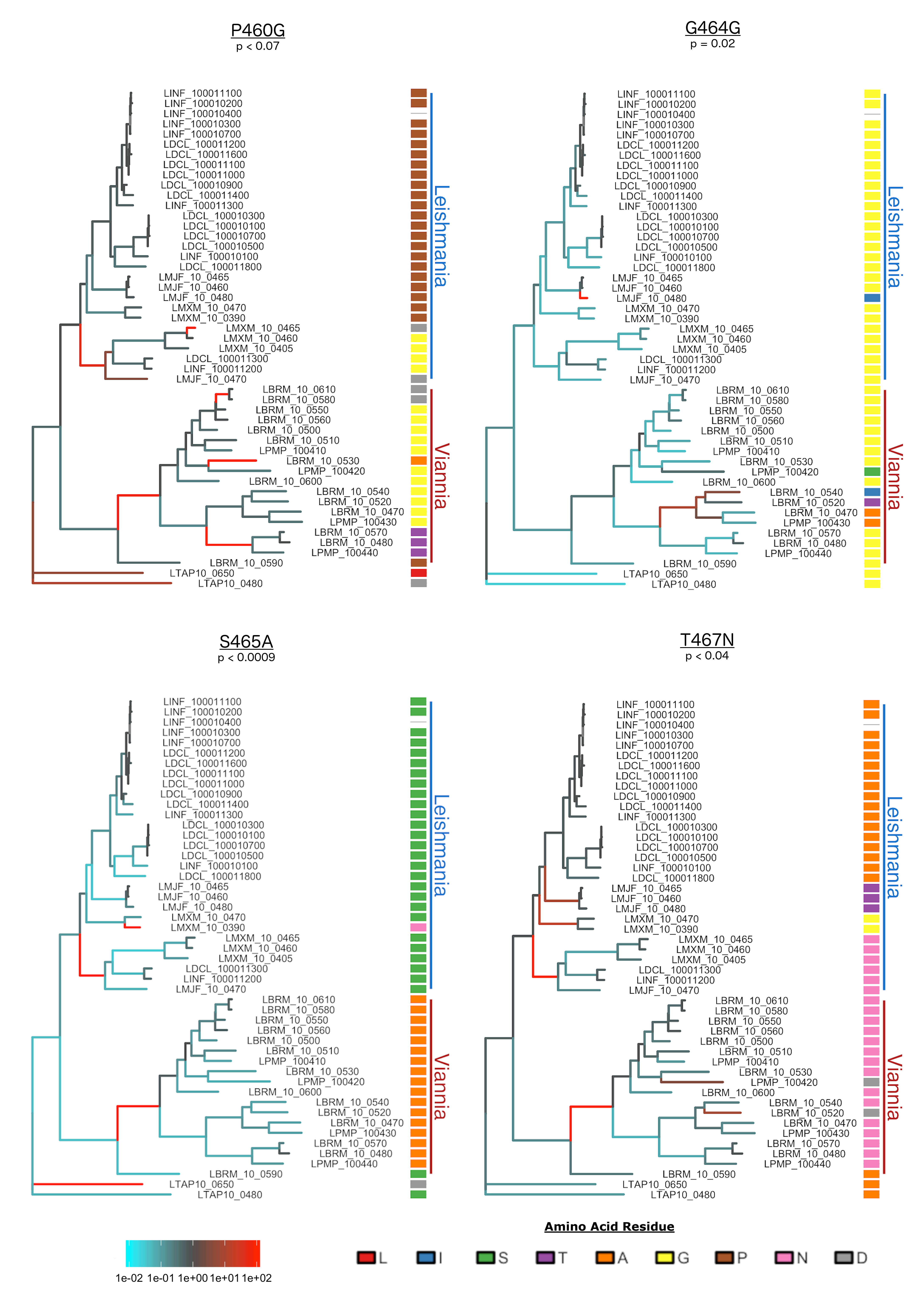


**S2 Fig. Episodic positive selection around the CXCL10 binding site on GP63 with labelled phylogenies.** Residues adjacent to the CXCL10 binding site demonstrate a pattern of exclusive mutation between the Leishmania and Viannia subgenera. The EBF generated by MEME was plotted onto the phylogenetic tree for positively selected residues (at a threshold of p<0.1) within 5 amino acids of the predicted CXCL10 binding site. The phylogenetic tree was rooted at the node of the most recent common ancestor of the two Sauroleishmania sequences identified. The amino acid residue for each sequence at the indicated position is plotted based on a multisequence alignment generated in ClustalOmega and used for both evolutionary tests above. MEME excludes identical sequences; therefore only 49 of 54 identified sequences are displayed in the phylogeny. P-values calculated based on likelihood ratio test statistic (LRT) as described in Murrell et al. 2012 [1]. Tip labels include species and genomic location. For species label, “LBRM” is *L. (V.) braziliensis* MHOM/BR/75/M2904, “LDCL” is *L. (L.) donovani* CL-SL, “LINF” *is L. (L.) infantum* JPCM5, “LMJF” is *L. (L.) major* strain Friedlin, “LMXM” is *L. (L.) mexicana* MHOM/GT/2001/U1103, “LPMP” is *L. (V.) panamensis* strain MHOM/PA/94/PSC-1, and “LTAP” is *L. tarentolae* Parrot-TarII.

Reference

1. Murrell B, Wertheim JO, Moola S, Weighill T, Scheffler K, Kosakovsky Pond SL. Detecting individual sites subject to episodic diversifying selection. PLoS Genet. 2012;8(7):e1002764. doi: 10.1371/journal.pgen.1002764. PubMed PMID: 22807683; PubMed Central PMCID: PMCPMC3395634.
