# Supplementary material for: Variation in Leishmania chemokine suppression driven by diversification of the GP63 virulence factor
Source: PLoS Negl Trop Dis. 2021 Oct 28;15(10):e0009224. doi: 10.1371/journal.pntd.0009224 (PMC8577781; doi:10.1371/journal.pntd.0009224)
Supplement: S3 Fig — As gp63 is present in a tandem array with variable copy number, we tested for associations of the proportion of the D463 allele with lesion number (A) or lesion type (B). Clinical characteristics were obtained from Patino et al. (2020) and Patino et al. (2020) [46,47]. Lesion number greater than one was used as a biological proxy for metastatic spread of infection. L. (V.) braziliensis isolates are colored in teal and L. (V.) panamensis isolates are colored in yellow. P-values obtained by two-tailed Mann-Whitney rank order test. To calculate the proportion of D463 allele: short read sequences from 7 L. (V.) braziliensis and 19 L. (V.) panamensis isolates were obtained from Patino et al. (2020) and Patino et al. (2020) [46–47] and aligned to the L. (V.) panamensis PSC-1 reference genome. We then quantified the number of reads carrying the amino acid allele (N, D, A, or T) at gp63 position 463. The read number was further normalized to total read depth of each sample with a scale factor of 10000. Finally, the number of normalized D reads was divided by the normalized total gp63 reads to get the proportion of the D463 allele. (DOCX) [file pntd.0009224.s005.docx]

*
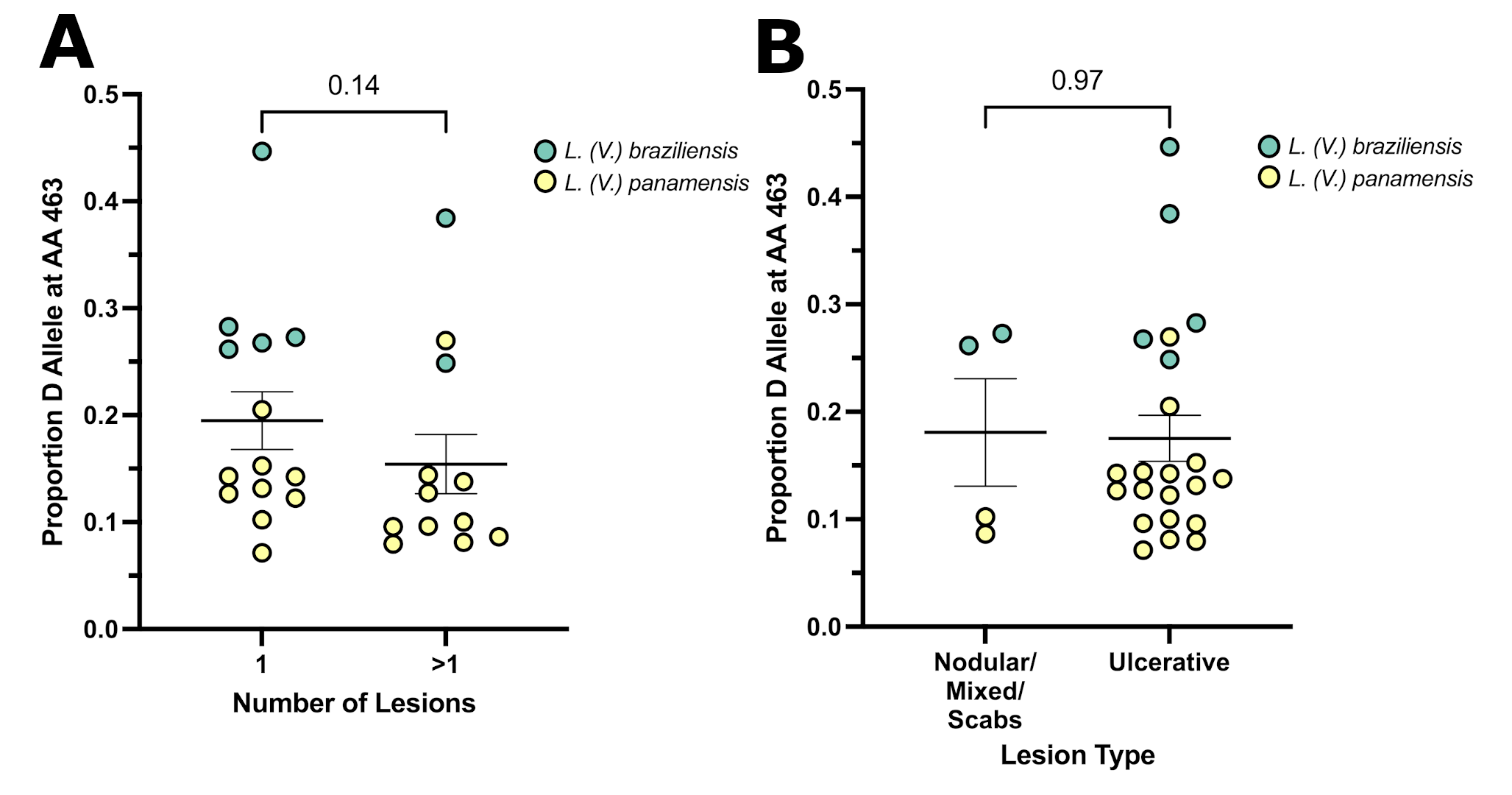
*

**S3 Fig. Association of CXCL10 binding D463 allele with clinical phenotypes of lesion number and lesion type.** As *gp63* is present in a tandem array with variable copy number, we tested for associations of the proportion of the D463 allele with lesion number (A) or lesion type (B). Clinical characteristics were obtained from Patino et al. (2020) and Patino et al. (2020) [1,2]. Lesion number greater than one was used as a biological proxy for metastatic spread of infection. *L. (V.) braziliensis* isolates are colored in teal and *L. (V.) panamensis* isolates are colored in yellow. P-values obtained by two-tailed Mann-Whitney rank order test. To calculate the proportion of D463 allele: short read sequences from 7 *L. (V.) braziliensis* and 19 *L. (V.) panamensis* were obtained from Patino et al. (2020) and Patino et al. (2020) [1,2] and aligned to the *L. (V.) panamensis* PSC-1 reference genome. We then quantified the number of reads carrying the amino acid allele (N, D, A, or T) at *gp63* position 463. The read number was further normalized to total read depth of each sample with a scale factor of 10000. Finally, the number of normalized D reads was divided by the normalized total *gp63* reads to get the proportion of the D463 allele.

References

1. Patino LH, Munoz M, Cruz-Saavedra L, Muskus C, Ramirez JD. Genomic Diversification, Structural Plasticity, and Hybridization in Leishmania (Viannia) braziliensis. Front Cell Infect Microbiol. 2020;10:582192. Epub 2020/11/13. doi: 10.3389/fcimb.2020.582192. PubMed PMID: 33178631; PubMed Central PMCID: PMCPMC7596589.

2. Patino LH, Munoz M, Muskus C, Mendez C, Ramirez JD. Intraspecific Genomic Divergence and Minor Structural Variations in Leishmania (Viannia) panamensis. Genes (Basel). 2020;11(3). Epub 2020/03/04. doi: 10.3390/genes11030252. PubMed PMID: 32120946; PubMed Central PMCID: PMCPMC7140786.
